# CD81 Aggravates Ovarian Cancer Progression via p‐Cresyl Sulfate‐Mediated Mitophagy in Tim4^+^ Tumour‐Associated Macrophages

**DOI:** 10.1111/jcmm.70701

**Published:** 2025-07-02

**Authors:** Jiali Ni, Xiaoying Li, Yue Wu, Xiaodi Tu, Xinxin Zhang, Lu Wang, Hao Xie, Yayi Hou, Huan Dou, Shuli Zhao

**Affiliations:** ^1^ Division of Immunology, The State Key Laboratory of Pharmaceutical Biotechnology, Medical School Nanjing University Nanjing China; ^2^ Jiangsu Key Laboratory of Molecular Medicine Nanjing China; ^3^ School of Life Science and Technology, The Key Laboratory of Developmental Genes and Human Disease Southeast University Nanjing China; ^4^ General Clinical Research Center, Nanjing First Hospital Nanjing Medical University Nanjing China

**Keywords:** CD81, ovarian cancer, PCS, TAM

## Abstract

Ovarian cancer (OC) is characterised by widespread peritoneal metastasis. Tetraspanin CD81 is predominantly located at the cellular membrane and exhibits inconsistent roles in tumour progression. However, its precise function in OC remains unclear. We found that CD81 expression was significantly elevated in tumour tissues from OC patients with poor prognosis, and it directly promoted proliferation, and migration of OC cells. Stable knock‐down of CD81 expression ameliorated disease progression in a murine model of OC and induced metabolic responses in OC cells. Metabolomics and mass spectrometry identified the protein‐bound toxin p‐cresyl sulfate (PCS) as a key metabolite regulated by the CD81‐FAK signalling axis. One aspect is that PCS promoted the growth of OC cells. Furthermore, tumour‐derived PCS combined with Cdh1 to enhance Bnip3‐dependent mitophagy activity of Tim4 positive tumour‐associated macrophages (TAMs). Intraperitoneal injection of PCS reversed the therapeutic effects observed following CD81 knock‐down; the mitophagy of reprogrammed Tim4^+^ TAMs was also promoted, accompanied by alterations in antitumor immunity. In summary, we elucidated CD81 prompted Tim4^+^ TAMs mitophagy to induce OC progression via FAK/PCS/Cdh1 pathway, deepen our understanding of OC pathogenesis.

## 1 Introduction

Ovarian cancer (OC) is one of the leading causes of cancer death in global women [1]. An estimated 314,000 new ovarian cancer cases and 207,000 deaths occur globally each year [2]. In China alone, over 39,000 women die from ovarian cancer in 2022, ranking as the second deadliest gynaecological cancer [3, 4]. The 5‐year survival rate of ovarian cancer patients in China is 40%, much lower than the 50.8% rate observed in the United States [5, 6]. Drug resistance, coupled with genetic mutations, hormonal influences and environmental factors contribute to the diverse manifestations and diagnostic challenges associated with ovarian cancer [7]. Clinical statistics reveal that 62%–75% of ovarian cancer patients exhibit intraperitoneal metastases–substantially higher than rates seen in other cancers. Moreover, 75% of recurrent disease develops intraperitoneally [8, 9]. Due to a lack of anatomical barriers, ovarian cancer cells can easily detach from primary tumours located within the epithelium of either the ovary or fallopian tube and subsequently access peritoneal fluid [10]. Ovarian cancer cells diffuse throughout the peritoneal cavity and rapidly develop into disease progression, commonly presenting with symptoms like small bowel obstruction, ascites, tumour‐related pain and malnutrition [11]. Thus, a comprehensive understanding of ovarian cancer pathogenesis is urgently needed.

Tetraspanin CD81, originally designated as anti‐proliferative antibody recognition target molecule‐1 (TAPA‐1), is broadly localised to the cell membrane. It serves as a scaffold that facilitates interactions with signalling molecules, thereby regulating cell migration and invasion [12, 13]. Consequently, CD81 has been implicated in cancer progression [14]. For instance, knockout of CD81 in the osteosarcoma cell line 143B reduced tumour formation and lung metastasis in mice [15]. In acute lymphoblastic leukaemia, CD81 knock‐down promoted chemosensitivity, reduced cellular adhesion and disrupted in vivo bone marrow homing and engraftment [16]. Indeed, membrane expression level of CD81 was associated with a worse prognosis in a cohort of 134 acute myeloid leukaemia patients [17]. Recently, CD81 has also emerged as a novel immunotherapeutic target in human breast cancer [18, 19]. Interestingly, the role of CD81 in the development of several other cancers is strikingly different [20]. Type II phosphoinositide 4‐kinase (PI4KII) is reported to interact with CD81, remodel actin cytoskeleton and inhibit human hepatocellular carcinoma (HCC) cell motility [21]. Conversely, in gastric cancer cells, CD81 had anti‐proliferative functions through inhibiting the phosphorylation of mitogen‐activated protein kinase 14 (Mapk14) [22]. Beyond its direct cellular functions, CD81 also modulates the immune environment with tumour hosts to mediately involve cancer progression, such as impairing the suppressive ability of T regulatory cells (Tregs) and myeloid‐derived suppressor cells (MDSCs) in tumour‐bearing mice [23]. In ovarian cancer (OC), several studies have identified CD81 as an exosome marker, representing the release of exosomes from OC cells [24, 25]. However, our understanding regarding the role of CD81 in OC disease progression remains limited.

Cell metabolic changes are recognised as a hallmark of OC progression, and some metabolites play important roles in regulating the tumour microenvironment [26, 27]. p‐Cresyl Sulfate (PCS) is a prototype protein‐bound uremic toxin derived from aromatic amino acids, such as tyrosine and phenylalanine [28]. The toxicity of PCS has been verified in various diseases, including neuroinflammation, heart failure and children autism [29, 30, 31]. Emerging evidence also suggests that PCS possesses potent cancer‐promoting activity. For example, PCS increased intracellular ROS to trigger bladder cancer cell migration, and promoted the proliferation and migration of clear cell renal cell carcinoma via microRNA‐21/HIF‐1α axis [32, 33]. However, the regulatory contribution of PCS in OC remains unexplored.

In our study, we identified CD81 as a potential biomarker for OC. CD81 promoted the production of PCS, which not only regulated tumour growth but also enhanced the mitophagy of T cell immunoglobulin and mucin domain containing 4 (Tim4) positive tumour‐associated macrophages (TAMs). This cascade thus disrupted the peritoneal immune microenvironment in the murine model of OC. In summary, our study indicated that CD81 was a potential pathogenic target for OC, and its regulation of the crosstalk between cancer cells and TAMs was crucial in tumours.

## 2 Methods and Materials

### 2.1 Clinical Samples

Thirty‐four cancer specimens from patients who diagnosed with ovarian cancer in Nanjing First Hospital (Nanjing, China) were included in our study. All the studies were approved by the ethics committee of Nanjing First Hospital (KY20210628‐9), and informed consent was obtained from each investigator. Their clinical information and clinicopathological features were listed at Table S1.

### 2.2 Murine Model of Ovarian Cancer

Female C57BL/6 mice (age, 6–8 weeks old) were purchased from Cavens Biotechnology Co. Ltd. (Nanjing, China) and were maintained under specific pathogen‐free (SPF) conditions at a light–dark cycle. Mice were acclimatised in housing conditions for at least 1 week. Mice experiments were approved by the Institutional Animal Care and Use Committee of Nanjing First Hospital, Nanjing Medical University (DWLL20220719‐4).

All mice were randomly divided into different groups. NC ID8 cells or sh‐CD81 ID8 cells (5 × 10^6^ cells/mouse in 200 μL PBS) were intraperitoneal administered into mice respectively to establish abdominal transferred tumour models. After 8 weeks, mice were eventually euthanized by asphyxiation via carbon dioxide. Meanwhile, PCS (CAS No. 91978‐69‐7, Cat. NO.: HY‐111431A, NoMedChemExpress, NJ, USA) was intraperitoneal injection at 100 mg/kg/day in the final 4 weeks. Besides, hypodermics of these cells (1 × 10^7^ cells/mouse in 200 μL PBS) were used to establish subcutaneous tumour models, and the duration of the model was 12 weeks.

### 2.3 Cell Lines and Cell Culture

Human OC cell lines A2780 (RRID: CVCL_0134) and SKOV3 (RRID: CVCL_0532) cells, and murine OC cell line ID8 (RRID: CVCL_IU14) cells were kindly provided by Dr. Zhao from Nanjing First Hospital. The cancer cells were cultured in dulbecco's modified eagle medium (DMEM; Gibico, Grand Island, NY, USA) supplemented with 10% foetal bovine serum (FBS; Gibco).

Lentivirus‐short hairpin RNA (shRNA) system was delivered into ID8 cells to downregulate CD81 expression [34]. The lentivirus transfer plasmid PHY‐315 (U6‐MCS‐CMV‐luciferase‐PGK‐puro, 9954 bp) was used and the sequences of CD81 shRNA were as follows: 5′‐GTACCTCATTGGAATTGCA‐3′ (Zebrafish Biotech, Nanjing, China). After puromycin pressure screening and other steps [35], the stably transfected cell line (sh‐CD81 ID8 cells) and negative control cells (NC ID8 cells) were established.

Murine macrophage cell line RAW264.7 (RRID: CVCL_0493) cells was purchased from Cell Bank of Chinese Academy of Sciences, and was cultured in DMEM with 10% FBS. To simulate the tumour environment in vitro, cell culture supernatant from ID8 cells was supplemented into the culture system of RAW264.7 cells (1:1 with fresh DMEM with 10% FBS), called tumour‐conditioned RAW264.7 cells to overexpress Tim4 for our experiments. All cells were grown in a humidified incubator at 37°C in a humidified atmosphere of 5% CO_2_. All experiments were performed with mycoplasma‐free cells.

### 2.4 Isolation of Tim4^+^ TAMs

Sacrificed mice were disinfected in 75% ethanol for 5–10 min, and then were fixed on the dissection table. We used scissors and tweezers to carefully cut the abdomen skin layer and completely separate the skin layer from the muscular layer, and used a syringe to aspirate ascites from the murine model of ovarian cancer. Aseptic operations should be maintained at all times. Ascites was centrifuged at 300 *g* for 5 min, and resuspended in Ammonium‐Chloride‐Potassium (ACK) Lysing Buffer for 2 min to remove red blood cells. After termination of lysis with phosphate buffered saline (PBS) and centrifugation washing, peritoneal cell precipitation was resuspended in DMEM with 10% FBS and 1% penicillin–streptomycin. Tim4^+^ primary peritoneal TAMs were isolated by APC‐conjugated‐Tim4 antibody (Cat. No.: 130021, BioLegend, CA, USA) staining and anti‐APC microbeads (Cat. No.:130–090‐855, Miltenyi Biotec, BG Germany) pulling according to the manufacturer's instructions. Tim4^+^ TAMs were purified and used for RNA sequencing, mitophagy‐related detections, and in vitro cultured with PCS treatment (30 μg/mL).

### 2.5 Non‐Targeted Metabolomic Analysis

Ultra high performance liquid chromatography‐orbitrap exploris‐mass spectrometry (UHPLC‐OE‐MS) performed by Biotree Biotech Co. Ltd. (Shanghai, China) was used for non‐targeted metabolomic analysis [36]. Primary NC ID8 cells and sh‐CD81 ID8 cells were collected and their metabolites were extracted. The detailed experimental procedures were described in the Supporting Information.

### 2.6 Detection of PCS Through Multiple Reaction Monitoring (MRM) Mass Spectrometry

Different samples were collected to detect the concentration of PCS, including ascites, cells and tumour culture supernatants. We designed the method of detecting PCS, and the detailed experimental conditions was listed in the Supporting Information.

### 2.7 Mitophagy‐Associated Flow Cytometry Assays

We first obtained peritoneal cells of mice as shown in the preceding step. After staining of macrophage‐associated surface antibodies for 30 min, cells were washed and incubated with mitochondria‐related reagent for 30 min at 37°C in serum‐free DMEM medium. MitoSOX (5 μM) (Cat. No.: M36005, Thermo Fisher Scientific, MA, USA) was used to measure mitochondrial ROS. Similarly, MitoTracker Green (100 μM) (Cat. No.: C1048, Beyotime, Shanghai, China) was used to catch the active mitochondria and MitoTracker Deep Red (100 μM) (Cat. No.: C1032, Beyotime) was used to detect mitochondrial membrane potential. Finally, the stained cells were washed with PBS and then detected by flow cytometry.

### 2.8 Molecular Docking

The crystal structure of Cdh1 protein (PDB ID: 3QRB Chain A) was obtained from Protein Data Bank (PDB), and it was processed respectively using Schrödinger's Protein Preparation Wizard module (NY, USA), including: protein preprocess, regenerate states of native ligand, H‐bond assignment optimisation, protein energy minimisation and remove waters. The 2D structure file of PCS was processed using the LigPrep module in Schrödinger and all its 3D chiral conformations were generated. Then, the SiteMap module in Schrödinger was used to predict the optimal binding site, and then the Receptor Grid Generation module in Schrödinger was used to set the most suitable enclosing box to perfectly wrap the predicted binding site, and on this basis, the active site of Cdh1 protein was acquired. The treated ligand PCS was molecularly docked with the active site of the Cdh1 protein. The effect of solvent on the docking efficiency was evaluated by calculating MM‐GBSA (Molecular Mechanics‐Generalised Born Surface Area, MM‐GBSA dG Bind).

### 2.9 RNA Sequencing and GO, KEGG Analysis

Before read mapping, clean reads were obtained from the raw reads by removing the adaptor sequences and low‐quality reads. The clean reads were then aligned to human genome (GRCh38_Ensembl104) using the Hisat2. HTseq was used to get gene counts and FPKM method was used to determine the gene expression. We applied DESeq2 algorithm to filter the differentially expressed genes, after the significant analysis, *p*‐value were subjected to the following criteria: Fold Change > 2 or < 0.5; *p*‐value < 0.05. Gene ontology (GO) analysis was performed, and we downloaded the GO annotations from NCBI, UniProt and the Gene Ontology. Fisher's exact test was applied to identify the significant GO categories (*p*‐value < 0.05). Pathway analysis was used to find out the significant pathway of the differentially expressed genes according to KEGG database. We turn to the Fisher's exact test to select the significant pathway, and the threshold of significance was defined by *p*‐value < 0.05.

### 2.10 Statistical Analysis

Differences between the two treatment groups were analysed by the Student's *t*‐test, and comparisons among different groups were made by one‐way analysis of variance (ANOVA). A *p*‐value lower than 0.05 (*p* < 0.05) was considered statistically significant. All the statistical analyses were conducted using the GraphPad Prism 9 software (GraphPad Software Inc., CA, USA). All experiments were performed at least thrice.

Other materials and methods were described in the Supporting Information.

## 3 Results

### 3.1 OC Patients With High CD81 Expression Exhibited Poor Survival

**FIGURE 1:**
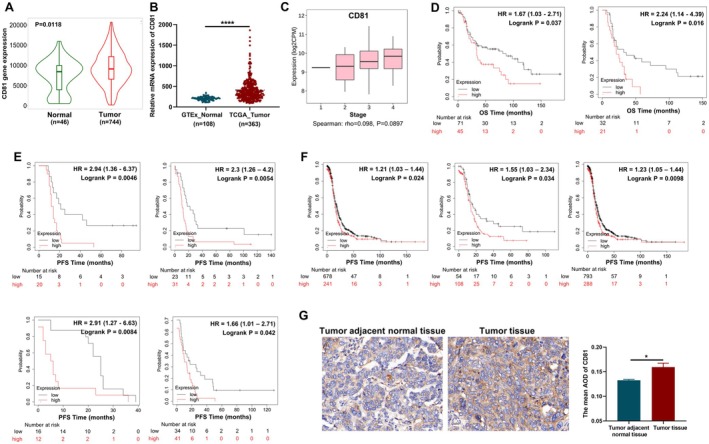
OC patients with high CD81 expression exhibited poor survival. (A) The expression of CD81 in OC tissues and normal ovarian tissues from TNMplot database. (B) mRNA expression of CD81 in OC tissues from TCGA database and normal ovarian tissues from GTEx database. (C) CD81 expression tendency in OC at different clinical stages from TISIDB database. (D) OS survival curves of CD81 expression in OC from GSE3149 and GSE18520 datasets. (E) PFS survival curves of CD81 expression in OC from GSE15622, GSE30161, GSE51373 and GSE63885 datasets. (F) PFS survival curves of CD81 expression for the OC patients with advanced cancer (Stage III–IV). (G) Immunohistochemical staining of CD81 in the tumour adjacent normal tissues and tumour tissues from OC patients, and the relative expression of CD81 was quantified by calculating the mean AOD. Data represent the mean scores±standard error of the mean (SEM). *p < 0.05, ****p < 0.0001.

Firstly, we discovered that gene expression of CD81 was elevated in OC tissues compared to normal tissues in the TNMplot database (Figure 1A). In the integration of TCGA and GTEx database, CD81 expression was significantly higher in OC samples than in normal tissues (Figure 1B). To evaluate the correlation between CD81 and disease progression, we used TISIDB database and found that CD81 expression showed an upward trend with cancer staging, but without significance (*p* = 0.0897, Figure 1C). The prognostic significance of CD81 in OC patients was analysed by Kaplan–Meier plotter. The overall survival (OS) of patients was curtailed obviously with high expression of CD81 (Figure 1D). Overexpression of CD81 was also markedly associated with poor progression‐free survival (PFS) in OC patients from various datasets (Figure 1E). In addition, the survival curves of OC patients with advanced cancer (stage III‐IV) showed the short PFS times along with high expression of CD81 (Figure 1F). We further analysed the protein expression of CD81 in tumour adjacent normal tissues and OC tissues from human samples through immunohistochemistry. As shown in Figure 1G, the average optical density (AOD) of CD81 signal indicated the expression of CD81, and the AOD value of tumour tissues was higher than that of tumour adjacent normal tissues. Interestingly, the clinical features were not associated with CD81 expression, indicating its overexpression in OC patients, regardless of age and clinical stage (Table S1). Collectively, these data suggested that CD81 was overexpressed in OC and could be the potential target for the treatment of the tumour with significant prognostic value.

### 3.2 CD81 Directly Modulated the Proliferation, Invasion and Migration of OC Cells

**FIGURE 2:**
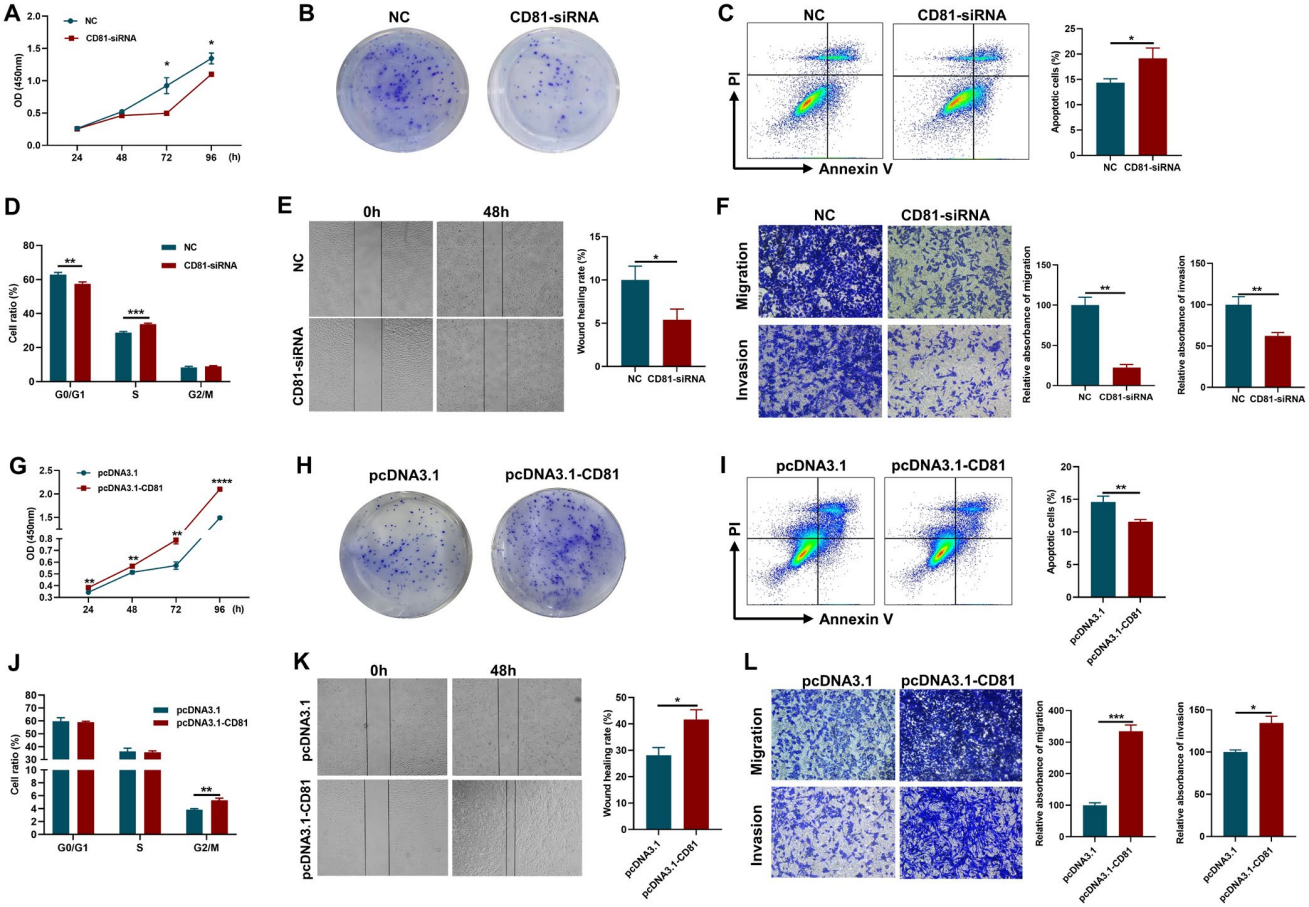
CD81 directly modulated proliferation, invasion and migration of OC cells. (A) CCK8 assay showed the effect of knocking down CD81 expression on cell growth in ID8 cells compared to the NC group (*n* = 4). (B) Representative images of cell colony formation showing the effect of knocking down CD81 expression on cell growth in ID8 cells compared to the NC group (*n* = 4). (C) Annexin V‐PI staining detected by flow cytometry showed the effects of knocking down CD81 expression on cell apoptosis in ID8 cells (*n* = 4). (D) PI staining detected by flow cytometry showed the effects of knocking down CD81 expression on the cell cycle in ID8 cells (*n* = 6). (E) Wound healing assay showed the effects of knocking down CD81 expression on cell migration in ID8 cells (*n* = 6). (F) Transwell assay cytometry showed the effects of knocking down CD81 expression on cell migration and invasion in ID8 cells, and representative images were shown (*n* = 3). (G) CCK8 assay showing the effect of CD81 overexpression on cell growth in ID8 cells compared to the NC group (*n* = 4). (H) Representative images of cell colony formation showed the effect of CD81 overexpression on cell growth in ID8 cells compared to the NC group (*n* = 4). (I) AnnexinV‐PI staining detected by flow cytometry showed the effects of CD81 overexpression on cell apoptosis in ID8 cells (*n* = 4). (J) PI staining detected by flow cytometry showed the effects of CD81 overexpression on the cell cycle in ID8 cells (*n* = 6). (K) Wound healing assay showed the effects of CD81 overexpression on cell migration in ID8 cells (*n* = 6). (L) Representative images of transwell assay cytometry showed the effects of CD81 overexpression on cell migration and invasion in ID8 cells (*n* = 3). Data represent the mean scores ± SEM. *p < 0.05, **p < 0.01, ***p < 0.001, ****p < 0.0001.

In order to gain an in‐depth insight into the biological functions of CD81 on OC cells, we employed three OC cell lines to elucidate the effect of differentially expressed CD81. For that, the transfection of CD81‐siRNA was validated by knocking down the mRNA and protein expression of CD81 in OC cells (Figure S1A,B). On the other hand, the pcDNA3.1‐CD81 plasmid was constructed and used to increase the mRNA and protein expression of CD81 in OC cells (Figure S1C,D). Based on the cell counting kit‐8 (CCK‐8) assays, knocked down expression of CD81 inhibited the proliferation of ID8 cells (Figure 2A), while its overexpression significantly promoted the proliferation of ID8 cells (Figure 2G). The colony formation ability of ID8 cells was suppressed visibly when decreasing the expression of CD81 (Figure 2B) and was enhanced in a CD81 expression‐dependent manner (Figure 2H). We also detected the apoptosis of OC cells by flow cytometry and found that the rate of apoptotic ID8 cells in the CD81‐siRNA group was significantly higher than in the NC group (Figure 2C); this phenomenon was reversed in the pcDNA3.1 and pcDNA3.1‐CD81 groups (Figure 2I). Cell cycle analysis showed that the knocked down expression of CD81 reduced the G0/G1 phase and increased the S phase, which led to S phase arrest in ID8 cells (Figure 2D). Then, the overexpression of CD81 increased the G2/M phase and expedited cell cycle progression in ID8 cells (Figure 2J). Moreover, CD81 was involved in the promotion of cell migration as assessed by the scratch assay (Figure 2E,K). Matrigel‐coated or non‐coated membranes were utilised to investigate the effect of CD81 on cell migration and invasion. The depletion of CD81 impaired the ability of ID8 cells to migrate and invade through the membranes of transwells (Figure 2F), while increased expression of CD81 had the opposite effect (Figure 2L).

**FIGURE 3:**
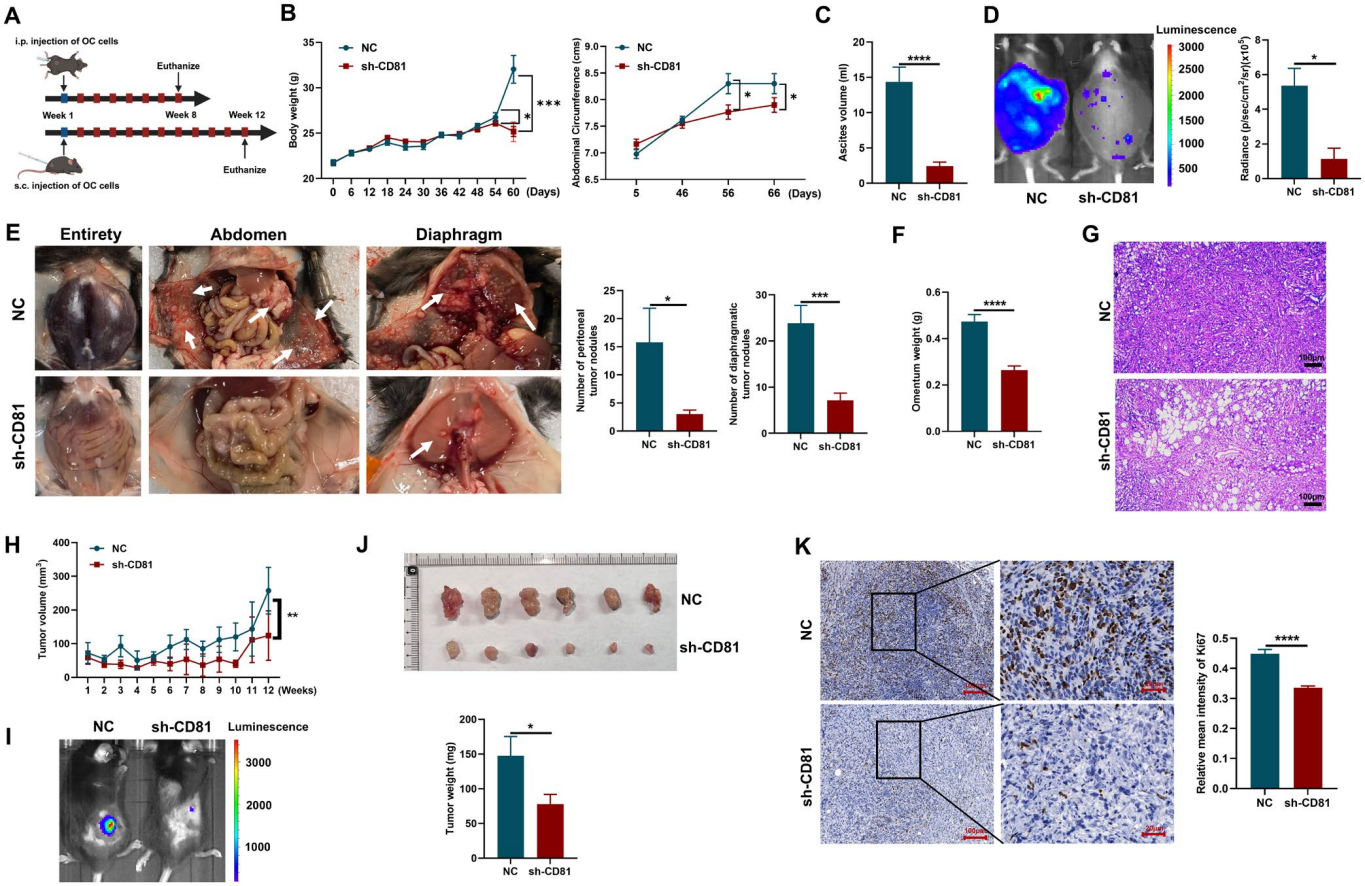
Stable knock‐down of CD81 expression in OC cells ameliorated tumourigenesis and metastasis in murine models of OC. (A) The flow chart of murine model. (B) The body weight and abdominal circumference changes in mice of NC and sh‐CD81 groups from the beginning to the end of peritoneal metastasis model construction. (C) The final volume of ascites in peritoneal metastasis murine tumour model of NC and sh‐CD81 groups. (D) Representative fluorescent images of mice using the in vivo imaging system in peritoneal metastasis murine tumour model of NC and sh‐CD81 groups; the statistical results of radiance were shown. (E) Representative images of the abdomen and diaphragm of peritoneal metastasis murine tumour model in NC and sh‐CD81 groups after sacrifice; the number of tumour nodules was quantified. (F) Omentum weight in peritoneal metastasis murine tumour model of NC and sh‐CD81 groups. (G) HE staining of the omentum in the peritoneal metastasis murine tumour model of NC and sh‐CD81 groups. (H) The body weight changes in the mice of NC and sh‐CD81 groups from the beginning to the end of establishing the subcutaneous model. (I) Representative fluorescent images of mice using the in vivo imaging system in the subcutaneous murine tumour model of NC and sh‐CD81 groups. (J) Representative images of the tumour of the subcutaneous murine tumour model in NC and sh‐CD81 groups after sacrifice, and the tumour weight was quantified. (K) Immunochemistry of Ki67 in the section of tumours from subcutaneous murine tumour model in NC and sh‐CD81 groups, and was quantified. The number of mice in the peritoneal metastasis tumour model was 10 per group, and the number of mice in the subcutaneous tumour model was 6 per group. Data represent the mean scores ± SEM. *p < 0.05, **p < 0.01, ***p < 0.001, ****p < 0.0001.

In addition, parallel detections were proceeded in two other OC cell lines A2780 and SKOV3 cells; the findings were consistent with those in ID8 cells (Figure S2 and Figure 3). Thus, CD81 had a comprehensive effect in vitro, including improving proliferation, migration and invasion and inhibiting apoptosis in OC cells.

### 3.3 Stable Knock‐Down of CD81 Expression in OC Cells Ameliorated Tumorigenesis and Metastasis in Murine Models of OC

We further explored the effect of CD81 in the murine models of OC (Figure 3A). The efficiency of stable knock‐down of CD81 expression in ID8 cells has been verified by Western blot and flow cytometry (Figure S4A,B). Next, we also detected some downstream signalling pathway‐related molecules of CD81 [37], and found that the phosphorylation of PI3K and Akt was altered in sh‐CD81 ID8 cells (Figure S4C). Mice in the sh‐CD81 group had lighter body weight and smaller abdominal circumference than the NC group during the terminal stage of the metastasis tumour model, indicating lower production of ascites in the sh‐CD81 group (Figure 3B,C). In vivo imaging exhibited that knock‐down of CD81 expression significantly relieved the tumour burden of advanced diffuse OC inside the abdominal cavity, indicating deceleration of cancer growth and metastasis (Figure 3D and Figure S5A). What's more, there were some clearly visible reductions in the number of peritoneal and diaphragmatic nodules in the mice from the sh‐CD81 group compared to the NC group (Figure 3E). The number of tumour nodules in the abdominal organs (liver, spleen and kidney) was also decreased by down‐regulating CD81 (Figure S5B). The omentum was recognised as the first site of metastasis for cancer cells in the abdominal cavity owing to physical proximity around the primary tumour, lack of barriers and wide connections [38, 39, 40]. The weight of the omentum was lighter while knocking down CD81 expression (Figure 3F). In the NC group, haematoxylin–eosin (HE) staining revealed residual vacuoles formed by adipose tissues and the infiltration of cancer cells into the omentum, and knocking down CD81 expression improved these phenomena (Figure 3G).

Furthermore, there was an observable reduction of tumour volume after knocking down CD81 expression of OC cells in making a subcutaneous tumour model (Figure 3H). Through fluorescence imaging, measuring size and weighing, we found that CD81 knock‐down absolutely inhibited tumour growth (Figure 3I,J). The expression of Ki67 was increased in the NC group, which showed greater proliferation of cancer cells compared to the sh‐CD81 group (Figure 3K). These results manifested that CD81 was a tumour promoter in OC.

### 3.4 The Growth of OC Cells Was Promoted by PCS Regulated Through CD8l‐FAK Signalling Pathway

**FIGURE 4:**
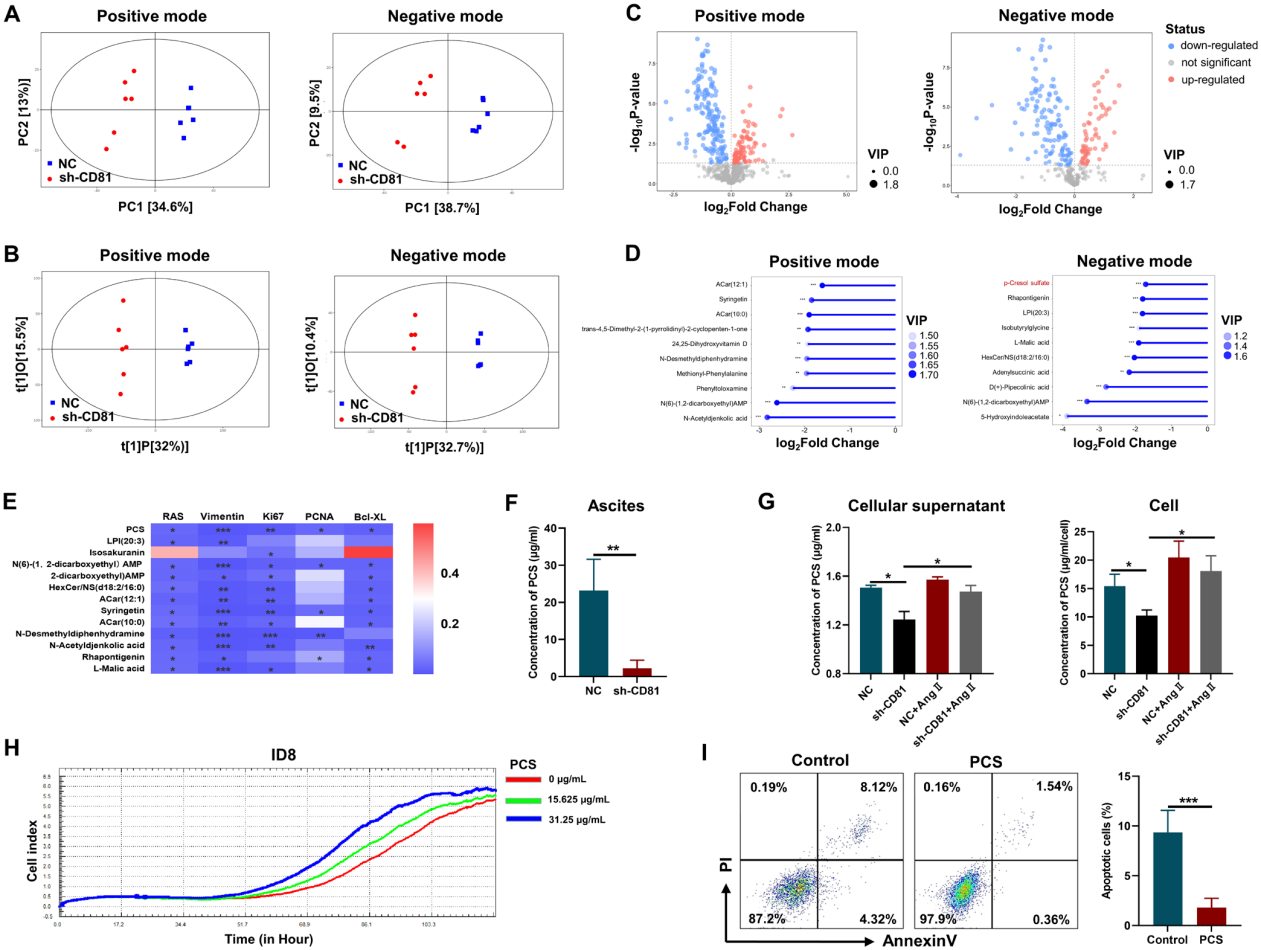
The growth of OC cells was promoted by PCS regulated through CD8l‐FAK signalling pathway. (A) Score scatter plot for PCA model in the positive and negative mode for obtaining the metabolomics data from NC and sh‐CD81 ID8 cells (*n* = 6). (B) Score scatter plot for orthogonal projections to latent structures‐discriminant analysis (OPLS‐DA) model in the positive and negative mode for obtaining the metabolomics data from NC and sh‐CD81 ID8 cells. (C) Volcano plot in the positive and negative mode for identifying the differential metabolites between NC and sh‐CD81 ID8 cells. (D) Matchstick analysis in the positive and negative mode for identifying the top 10 down‐regulated metabolites in sh‐CD81 ID8 cells compared to NC ID8 cells (*p < 0.05, **p < 0.01, ***p < 0.001). (E) Correlation analysis of significantly down‐regulated metabolites (*n* = 13, *p* < 0.001) with the relative mRNA expression of proliferation, apoptosis or CD81‐related molecules (*RAS*, *Vimentin*, *Ki67*, *PCNA* and *Bcl‐XL*). (F) Quantitative detection of PCS in the ascites detected by multiple reaction monitoring analysis in NC and sh‐CD81 groups (*n* = 4). (G) Quantitative detection of PCS in the cellular supernatant and cells detected by multiple reaction monitoring analysis from four groups: NC, sh‐CD81, NC + angiotensin II (Ang II), sh‐CD81+ Ang II (*n* = 4). (H) RTCA assay showed the effect of PCS with different concentrations on cell proliferation in ID8 cells. (I) Annexin V‐PI staining detected by flow cytometry showed the effects of PCS on apoptosis in ID8 cells. Data represent the mean scores ± SEM. *p < 0.05, **p < 0.01.

Some studies found that CD81 was a determinant of whole‐body energy homeostasis, which CD81 loss leads to a fuel switch toward carbohydrate oxidation and adipose tissue dysfunction [41]. Thus, we employed untargeted metabolomics via UHPLC‐OE‐MS to analyse the metabolites extracted from NC and sh‐CD81 ID8 cells. Principal component analysis (PCA) plots exhibited clear separation of the metabolites in sh‐CD81 ID8 cells from NC ID8 cells both in the positive and negative modes (Figure 4A). Orthogonal partial least squares‐discriminant analysis (OPLS‐DA) was also applied to obtain reliable differences of metabolites in the two groups that were distantly separated in the positive and negative modes (Figure 4B). Differential metabolites were identified by screening (variable importance in the projection, VIP > 1; *p*‐value < 0.05), shown in the volcano plot (Figure 4C). Next, we focused on the down‐regulated metabolites in sh‐CD81 ID8 cells and selected the top 10 metabolites with maximal enrichment (based on fold change) to display in the matchstick diagram, only one of them among these overlapped in the positive and negative modes (Figure 4D). To further determine the key metabolites, 13 metabolites with the most remarkable change (*p* < 0.001) were preferentially chosen to analyse the correlation with some major genes in NC and sh‐CD81 ID8 cells: proliferation‐related gene *Ki67* and *PCNA*, apoptosis‐related gene *Bcl‐XL*, metastasis‐related gene *Vimentin* and CD81 downstream signalling gene *RAS* (Figure 4E). The results showed that PCS was significantly correlated with all genes, and thus identified as the potential objective in ID8 cells for further substantiation. As shown in Figure 4D, the expression of PCS in the sh‐CD81 ID8 cells showed a marked decline in the negative mode. We designed a specific detection method to detect the level of PCS through MRM mass spectrometry. The results showed that the level of PCS was down‐regulated in the ascites from mice in the sh‐CD81 group than in the NC group, but how CD81 knock‐down regulated the production of PCS needs to be investigated further (Figure 4F). Focal adhesion kinase (FAK) is a downstream molecule in response to CD81 signalling during cell proliferation regulation in adipocyte progenitor cells [41]. CD81 knock‐down could inhibit the phosphorylation of FAK in ID8 cells (Figure S6). Furthermore, FAK activator, angiotensin II (Ang II), reversed the decrease in intracellular and secreted PCS levels in sh‐CD81 ID8 cells (Figure 4G). It demonstrated that tumour CD81modulate the production of PCS through the FAK signalling pathway.

The effect of PCS in OC is yet unknown. PCS directly promoted the proliferation of OC cells by CCK‐8 assay (Figure S6A). The facilitating effect of PCS on the growth of ID8 cells was dose‐dependent in real‐time cellular analysis (RTCA) (Figure 4H). The apoptosis of ID8 cells was inhibited after PCS treatment (Figure 4I). Thus, we found that PCS has cancer‐promoting activity in OC.

### 3.5 PCS Exhibited Potential in Regulating Tim4^+^ TAMs Survival

**FIGURE 5:**
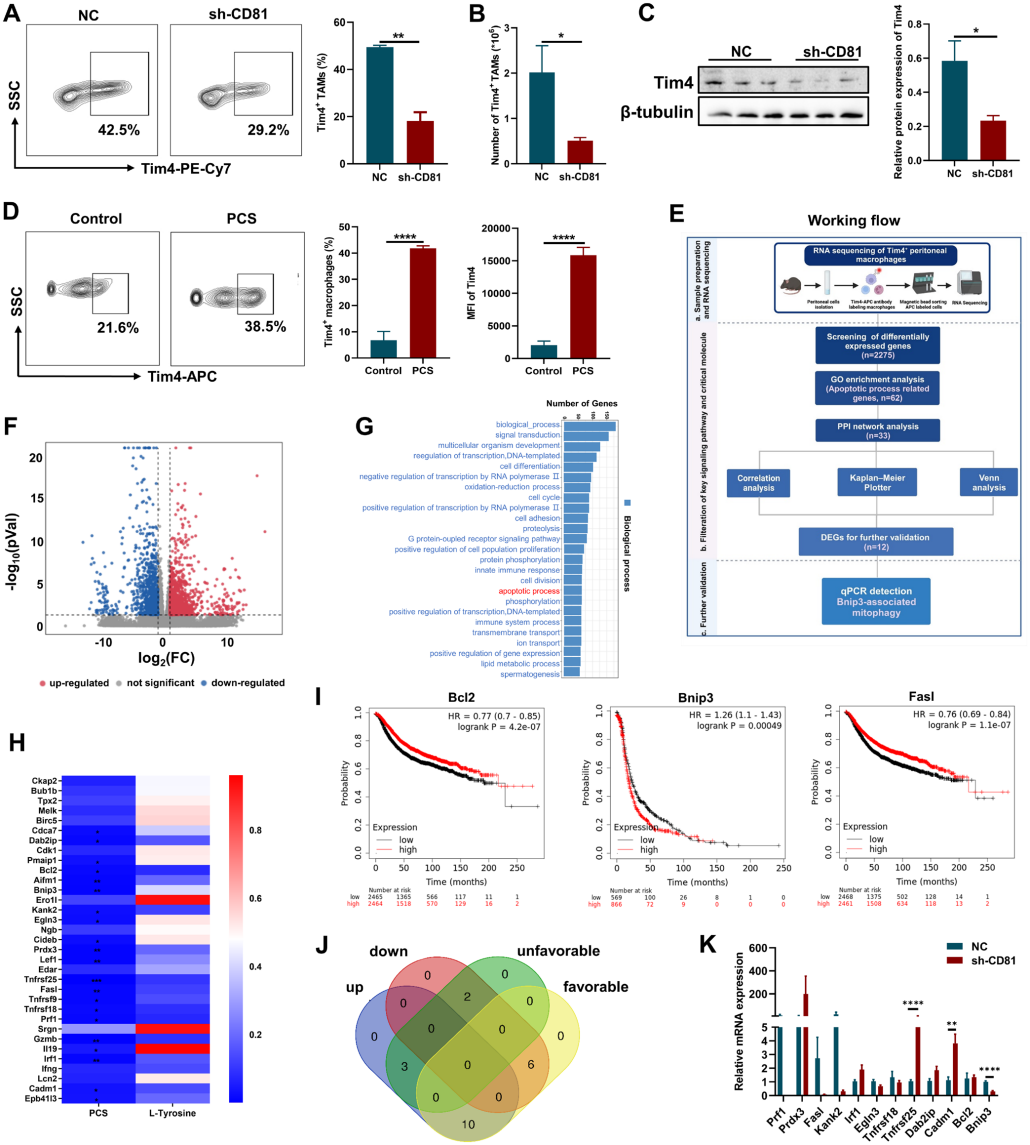
PCS exhibited potential in regulating Tim4^+^ TAMs survival. (A) Representative images of Tim4^+^ TAMs in peritoneal metastasis murine tumour model of NC and sh‐CD81 groups, and the percentage of Tim4^+^ TAMs was quantified (*n* = 6). (B) The number of Tim4^+^ TAMs in the peritoneal metastasis murine tumour model of NC and sh‐CD81 groups was quantified (*n* = 6). (C) Relative protein expression of Tim4 in RAW264.7 cells after co‐culture with TCM from NC and sh‐CD81 ID8 cells measured by Western blot (*n* = 3). (D) Representative images of Tim4^+^ macrophages in tumour‐conditioned RAW264.7 cells after PCS stimulation; the percentage of Tim4^+^ macrophages and MFI of Tim4 were quantified (*n* = 4). (E) The working flow of integrated analysis between RNA sequencing and database analysis. (F) Volcano plot for DEGs in the two groups of Tim4^+^ TAMs: Purified from peritoneal metastasis murine tumour model of NC and sh‐CD81 groups (*n* = 3). (G) GO enrichment analysis of DEPs, including biological process, arranged by the number oFf genes. (H) Correlation analysis of PCS and L‐Tyrosine with apoptotic process related molecules. (I) Venn diagram depicted the concordant proteins in DEPs from unfavourable/favourable and down‐/up‐regulated in OC. (J) Survival curves of three typical cell death‐associated molecules (Bcl2, Fasl and Bnip3) in OC. (K) Relative mRNA expression of 12 vital molecules for further validation in the ascites isolated from NC and sh‐CD81 ID8 cells measured by qPCR (*n* = 8). Data represent the mean scores ± SEM. *p < 0.05, **p < 0.01, ***p < 0.001, ****p < 0.0001.

Macrophages are the most abundant immune cells in the peritoneal fluid, playing an important role in OC metastasis [42, 43]. Specifically, Tim4 is a phosphatidylserine receptor expressed on macrophages and has been described as a specific marker for co‐labelling embryo‐derived TAMs [44]. In the murine model of OC, the absolute number of Tim4^+^ TAMs in the peritoneal fluid were increased with disease duration via local self‐expansion [45]. When stable knock‐down CD81 ameliorated the growth of ovarian cancer, it is worth paying attention to whether Tim4^+^ TAMs change. We then found that the proportion and number of Tim4^+^ TAMs (CD45^+^CD11b^+^F4/80^+^Tim4^+^) were both decreased when CD81 expression of OC cells was knocked down in the peritoneal metastasis murine tumour model, which were in accordance with the remission of tumours (Figure 5A,B). Thus, Tim4^+^ TAMs could give us a better understanding of CD81 in tumour‐promoting role in ovarian cancer.

For in vitro experiments, we used tumour conditional medium (TCM) from ID8 cells (NC or sh‐CD81 ID8‐TCM) to induce Tim4^+^ RAW264.7 cells (tumour‐conditioned RAW264.7 cells), and the overexpression of Tim4 protein has been verified after treatment (Figure S7A). We applied TCM from NC and sh‐CD81 ID8 cells to culture murine macrophages RAW264.7 cells in vitro, and the protein expression of Tim4 was reduced in the RAW264.7 cells co‐cultured with supernatant from sh‐CD81 ID8 cells (Figure 5C). Meanwhile, PCS also directly increased the proportion of Tim4^+^ cells and the mean fluorescence intensity (MFI) of Tim4 in the tumour‐conditioned RAW264.7 cells (Figure 5D). The protein expression of Tim4 was also elevated after PCS treatment (Figure S7B). Overall, it hinted that PCS might be the key mediator to transfer information between OC cells and Tim4^+^ TAMs through CD81 signalling, but the effect of tumour‐derived PCS on Tim4^+^ TAMs under CD81 signalling need to explore.

Furthermore, we purified Tim4^+^ TAMs from the peritoneal metastasis tumour models (NC and sh‐CD81 groups) for RNA sequencing. The method of integrated analysis was designed for further screening and assess the functional change in Tim4^+^ TAMs (Figure 5E). Over 90% Tim4‐positive cells were collected after sorting (Figure S7C). 2275 differentially expressed genes (DEGs) (fold change > 2 or < 0.5; *p* < 0.05) were identified between Tim4^+^ TAMs from two groups (Figure 5F). Gene ontology (GO) analysis was performed to elucidate the biological implications of these DEGs, and the apoptotic process was under intensive focus because of the decreased number of Tim4^+^ TAMs in the sh‐CD81 group (Figure 5G). The protein–protein interaction (PPI) network of 62 apoptosis‐related genes was visualised based on STRING analysis, at a minimum required interaction score of > 0.4 (medium confidence) (Figure S8A). Of the 33 interconnected genes which were selected for further filtration, the expression of 21 genes in the Tim4^+^ TAMs from the sh‐CD81 group were remarkably correlated with the level of PCS, but not with precursor substance of PCS, L‐tyrosine (Figure 5H). The survival curves of 21 genes in OC were also displayed, and most of them displayed the satisfactory prognostic value for OC, especially those associated with cell death, such as Bcl2, Fas ligand (Fasl), and BCL2/adenovirus E1B interacting protein 3 (Bnip3) (Figure 5I and Figure S8B). Subsequently, the 21 genes were divided into two subgroups, favourable or unfavourable for OC. We applied the conjoint analysis based on both the two subgroups and the alteration of gene expression in the Tim4^+^ TAMs from the sh‐CD81 group, and found that there were 12 intersectant genes, including 2 unfavourable down‐regulated genes and 10 favourable up‐regulated genes (Figure 5J). These 12 genes were ultimately verified by qPCR in the ascites isolated from NC and sh‐CD81 groups, and the expression of 3 genes were significantly altered in the sh‐CD81 group (Figure 5K). Among these, Bnip3 mRNA expression was one of most prominently decreased in the Tim4^+^ TAMs from the sh‐CD81 group. Bnip3 is localised to mitochondria and mediates mitochondrial depolarization and oxidative stress to regulate the process of mitophagy, contributing to the regulation of cell death [46]. Accordingly, we hypothesised that Bnip3‐dependant mitophagy regulated by PCS might be associated with Tim4^+^ TAMs survival.

### 3.6 PCS Enhanced the Mitophagy of Tim4^+^ Macrophages by Binding Cdh1

**FIGURE 6:**
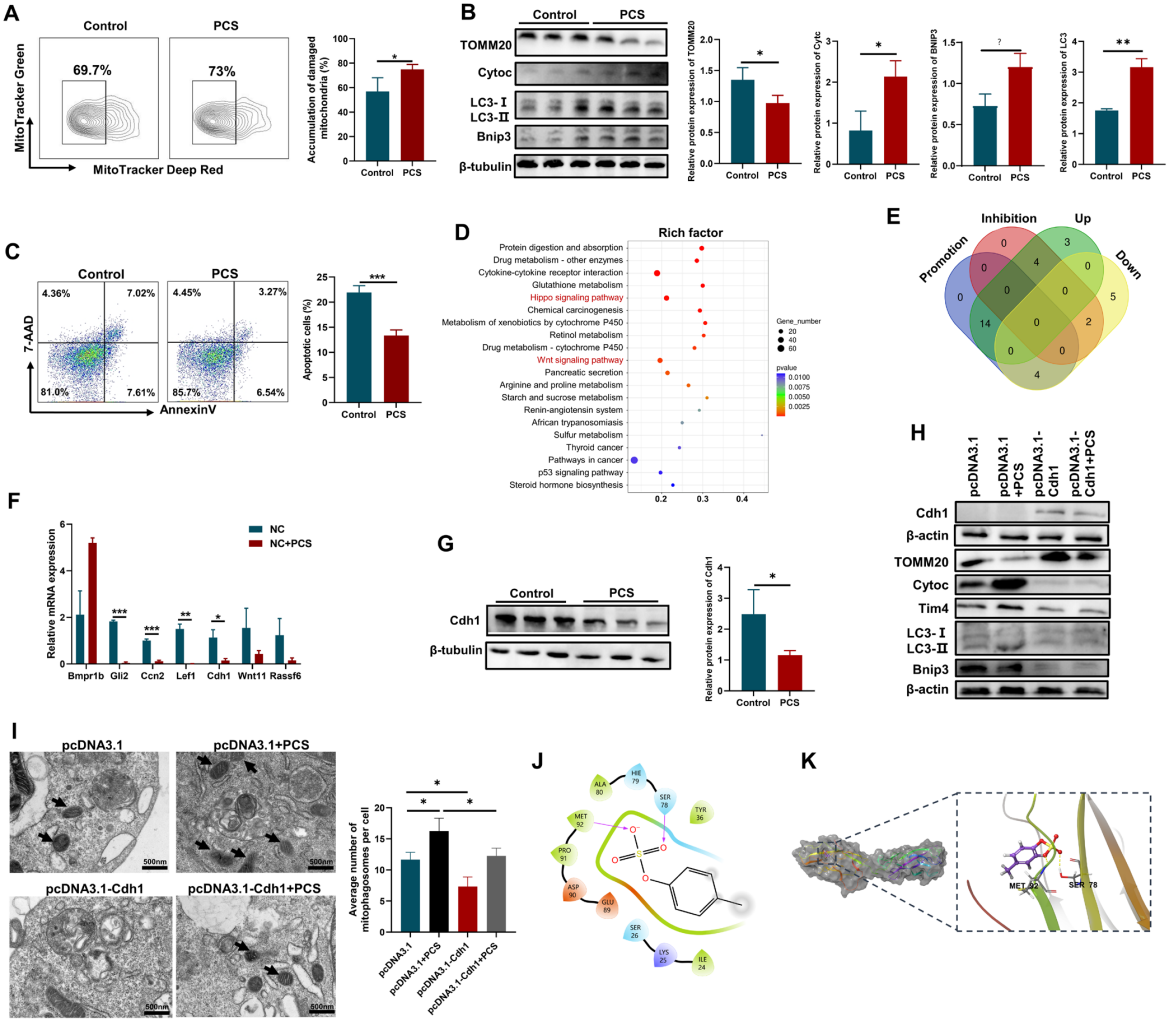
PCS enhanced the mitophagy of Tim4^+^ macrophages by binding Cdh1. (A) Tumour‐conditioned RAW264.7 cells were treated with PCS, and these cells were stained with MitoTracker Green and MitoTracker Deep Red to assess the damaged mitochondria by flow cytometry. The histograms and statistical results were depicted (*n* = 6). (B) Relative protein expression of mitophagy‐associated molecules in tumour‐conditioned RAW264.7 cells after PCS stimulation measured by Western blot (*n* = 3). (C) Annexin V‐PI staining detected by flow cytometry showed the effects of PCS on apoptosis in Tim4^+^ cells in tumour‐conditioned RAW264.7 cells (*n* = 6). (D) KEGG analysis of DEPs between two groups of Tim4^+^ TAMs (mentioned in Figure 5) arranged by rich factor. (E) Venn diagram depicted the concordant proteins in DEPs from Hippo signalling pathway from promoted/inhibited OC and down‐/up‐regulated proteins in OC. (F) Relative mRNA expression of 7 genes for further validation in primary Tim4^+^ TAMs from the murine model of OC after PCS stimulation in vitro measured by qPCR (*n* = 3). (G) Relative protein expression of Cdh1 for further validation in Tim4^+^ macrophages in tumour‐conditioned RAW264.7 cells after PCS stimulation measured by Western blot (*n* = 3). (H) Relative protein expression of mitophagy‐associated molecules in tumour‐conditioned RAW264.7 cells after different treatments: Transfecting pcDNA3.1/pcDNA3.1‐Cdh1, with/without PCS stimulation (*n* = 3). (I) Representative TEM pictures and statistical results of autophagosomes containing mitochondria in tumour‐conditioned RAW264.7 cells. Black arrows indicate mitophagosomes (*n* = 3). (J) The two‐dimensional binding model of PCS and Cdh1. (K) The three‐dimensional binding model of PCS on the molecular surface of Cdh1, yellow colour represented hydrogen bonds. Data represent the mean scores ± SEM. *p < 0.05, **p < 0.01, ***p < 0.001.

To further explore whether PCS could adapt mitophagy function in Tim4^+^ TAMs, we firstly investigated the accumulation of damaged mitochondria by MitoTracker Green staining, a mitochondrial mass indicator of living cells combined with MitoTracker Deep Red staining, an assay to measure mitochondrial membrane potential [45, 47, 48]. Figure 6A showed that PCS treatment caused accumulation of damaged mitochondria (MitoTracker Green^+^/MitoTracker Deep Red^lo^) in tumour‐conditioned RAW264.7 cells. Next, we detected the expression of mitophagy‐related proteins, including translocase of outer mitochondrial membrane 20 (TOMM20), mitochondrial cytochrome C (Cytoc), microtubule‐associated protein 1A/1B‐light chain 3 I/II (LC3‐I/II), and Bnip3 (Figure 6B). PCS inhibited the expression of TOMM20, and improved the expression of Bnip3, Cytoc as well as the conversion of LC3‐I to LC3‐II, which collectively emphasised the promotion of mitophagy in tumour‐conditioned RAW264.7 cells after PCS treatment. Then, the apoptosis of Tim4^+^ macrophages was apparently suppressed by PCS (Figure 6C). The data suggested that PCS regulated mitophagy in Tim4^+^ macrophages.

We continued to discover the underlying mechanisms of PCS on Tim4^+^ macrophages. Based on KEGG analysis of RNA sequencing, only 37 significant pathways were identified. Hippo signalling pathway with great variance has been reported in OC and has been selected as the intriguing pathway for further screening (Figure 6D). After the overlapping analysis based on literature retrieval and the results of RNA sequencing, 32 DEPs enriched in the Hippo signalling pathway comprised 4 down‐regulated genes with cancer‐promoting function and 4 up‐regulated genes with cancer‐inhibiting function (Figure 6E). In addition to one uncommon gene, the mRNA expression of the other 7 genes was detected by qPCR; and PCS significantly decreased the mRNA expression of *Cdh1*, *Ccn2*, *Gli2* and *Lef1* in the Tim4^+^ TAMs in vitro (Figure 6F). Cdh1 is a type of cell surface transmembrane glycoprotein [49]. Stieg et al. demonstrated that Cdh1 could bind to Parkin (an essential E3 ligase for ubiquitin‐mediated mitophagy), which manifested the effect of Cdh1 in the process of mitophagy [50]. We further detected the expression of Cdh1 in tumour‐conditioned RAW264.7 cells, and PCS inhibited the protein expression of Cdh1 (Figure 6G). Subsequently, the overexpression of Cdh1 inhibited the protein expression of Tim4 in tumour‐conditioned RAW264.7 cells and eliminated the elevated expression of Tim4 caused by PCS (Figure 6H). The enhanced mitophagy of RAW264.7 cells caused by PCS was reversed by the overexpression of Cdh1, suggesting that Cdh1 was the crucial molecule regulating mitophagy mediated by PCS (Figure 6H). The formation of mitophagosome signals the genesis of mitophagy. We found that PCS increased the number of mitophagosomes in tumour‐conditioned RAW264.7 cells, and the overexpression of Cdh1 decreased the number of mitophagosomes with or without PCS treatment (Figure 6I). Molecular docking was used to explore the concrete relationship of PCS and Cdh1. The results suggested that PCS directly bound to the surface of the active pocket of Cdh1 protein and formed a hydrophobic force via PRO91, MET92, ALA80, TYR36 and ILE24 residues of Cdh1 protein, specially forming a hydrogen bond through MET92 and SER78 residues (Figure 6J,K). The binding free energy (MM‐GBSA dG Bind) was estimated as −15.45 kcal/mol. Consequently, we speculated that PCS bound to Cdh1 to modulate the mitophagy of Tim4^+^ macrophages.

### 3.7 Mitigative Effect of Sh‐CD81 on OC Was Reversed by PCS

**FIGURE 7:**
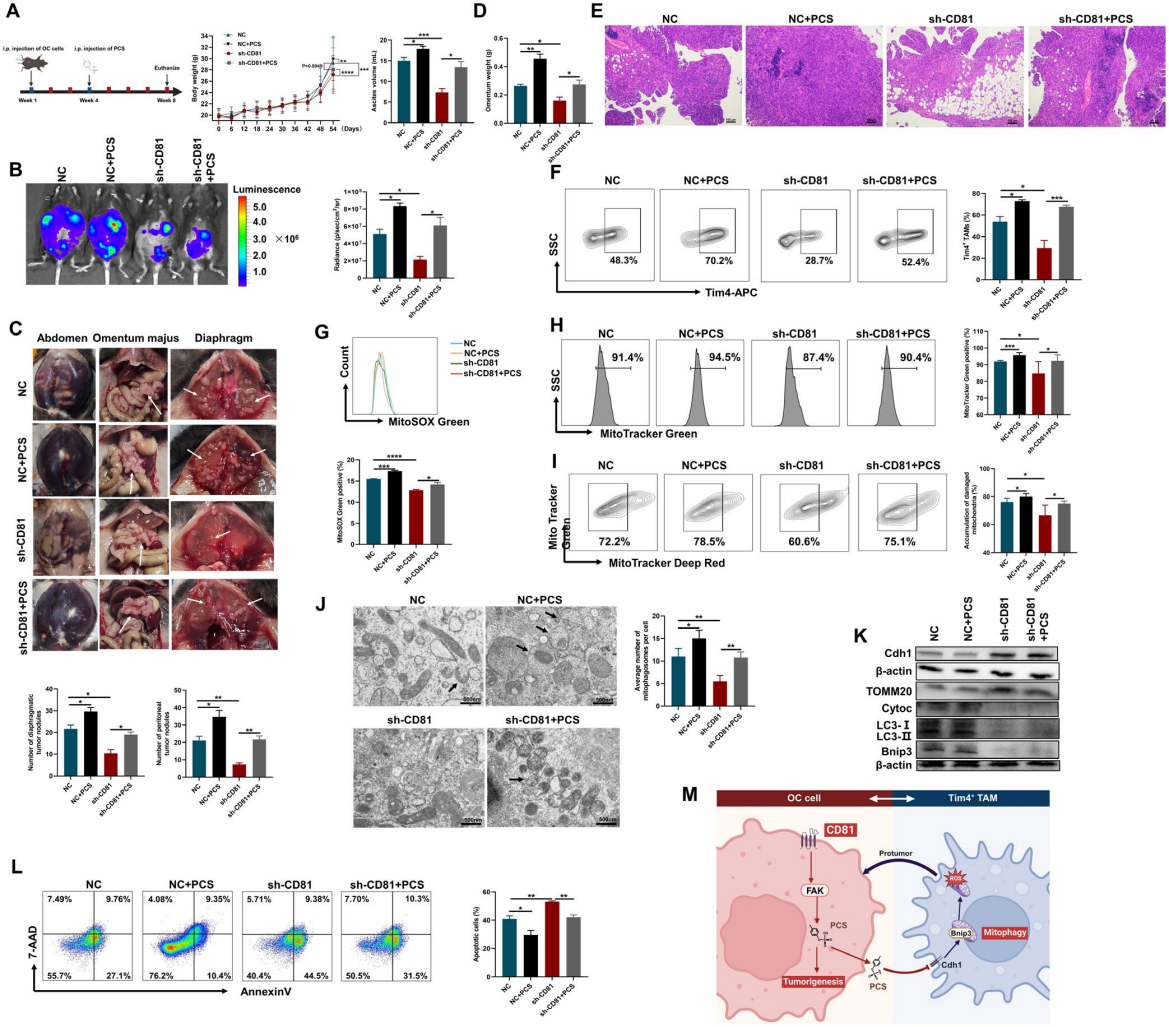
The mitigative effect of sh‐CD81 on OC was reversed by PCS. (A) The flow chart of establishing model, and the body weight changes in mice of four groups from the beginning to the end of constructing peritoneal metastasis model, and the final volume of ascites in the peritoneal metastasis murine tumour model of these four groups. (B) Representative fluorescent images of mice using the in vivo imaging system in four groups, and the statistical results of radiance were showed. (C) Representative images of the abdomen and diaphragm after sacrifice, and the number of tumour nodules was quantified. (D) The omentum weight in mice of the four groups. (E) HE staining of the omentum in peritoneal metastasis murine tumour model of the four groups. (F) Representative images of Tim4^+^ TAMs in four groups, and the percentage of Tim4^+^ TAMs was quantified. (G) Tim4^+^ TAMs from the four groups were stained with MitoSOX Green, as detected by flow cytometry to measure the production of superoxide by mitochondria. The histograms and statistical results were depicted. (H) Tim4^+^ TAMs from the four groups were stained with MitoTracker Green to detect the number of mitochondria. The histograms and statistical results were depicted. (I) Tim4^+^ TAMs from the four groups were stained with MitoTracker Green and MitoTracker Deep Red, and the representative images and statistical results were depicted. (J) Representative TEM pictures of autophagosomes containing mitochondria in Tim4^+^ TAMs from four groups. Black arrows indicate mitophagosomes. (K) Relative protein expression of mitophagy‐associated molecules in Tim4^+^ TAMs from four groups. (L) Annexin V‐PI staining detected by flow cytometry showed the effects of PCS on cell apoptosis in Tim4^+^ TAMs from four groups. *n* = 10 per group. (M) A schematic diagram showing how CD81 regulated tumour progression and immune environment. Data represent the mean scores ± SEM. *p < 0.05, **p < 0.01, ***p < 0.001, ****p < 0.0001.

As PCS contributed to OC cell growth and Tim4^+^ TAM survival, it was applied to the murine model (Figure 7A). Compared to the sh‐CD81 group, PCS administration increased the body weight and volume of ascites in the sh‐CD81 + PCS group (Figure 7A). Even knock‐down of CD81 expression in ID8 cells, the exacerbation of tumour progression mediated by PCS was showed by in vivo imaging, calculating the number of tumour nodules and HE staining of omentum (Figure 7B–E). Besides, the mice in the NC + PCS group had a more deteriorative tumour progression than the mice in the NC group, indicating that PCS as a toxin directly promoted the development of OC.

The proportion of Tim4^+^ TAMs was elevated in the sh‐CD81 + PCS group compared to the sh‐CD81 group (Figure 7F). In line with this result, the levels of mitochondria‐related ROS increased (Figure 7G). MitoTracker Green supported that CD81 knock‐down decreased the percentage of mitochondria in Tim4^+^ TAMs, whereas these changes were reversed by PCS administration (Figure 7H). The damaged mitochondria were accumulated in the PCS group, and CD81 knckdown alleviated the damage of mitochondria in Tim4^+^ TAMs (Figure 7I). Transmission electron microscopic observation revealed decreased number of mitophagosome in the Tim4^+^ TAMs in the sh‐CD81 group, and PCS promoted the production of mitophagosome (Figure 7J). The detection of Bnip3‐dependent mitophagy‐related proteins also indicated the function of PCS (Figure 7K). As expected, the apoptosis of Tim4^+^ TAMs were increased in the sh‐CD81 group, while PCS inhibited the production of apoptotic cells (Figure 7L).

Tim4^+^ TAMs could impair the proliferation of CD8^+^ T cells in peritoneal cavity and have protumor functions [51]. We also analysed the T cell phenotype in ID8 models. The proportion of immunosuppressive T regulatory (Treg) cells in ascites was decreased in the sh‐CD81 group, while PCS increased the accumulation of Treg cells (Figure S9A). The expression of programmed cell death protein 1 (PD‐1) in T cells exhibited same changes in the ascites from the four groups (Figure S9B). Hence, PCS regulated the T cell‐dependent antitumor responses through the mitophagy of Tim4^+^ TAMs to promote tumour progression.

## 4 Discussion

Herein, we provided a comprehensive illustration of the integrated cell–cell crosstalk between cancer cells and Tim4^+^ TAMs regulated by CD81 (Figure 7M). The dual effects of CD81 were both on OC cells and the peritoneal immune microenvironment. Silencing CD81 expression in OC cells directly inhibited their proliferation, invasion and migration, simultaneously suppressed mitophagy in Tim4^+^ TAMs in a murine model of ovarian cancer through inhibiting the production of PCS. Our study indicated the potential of CD81 as an effective therapeutic target for OC. Several studies have focused on anti‐CD81 antibodies treatment concerning tumour progression and metastasis [52], and therapies targeting CD81 could be developed for future cancer treatment. On the other side, CD81 is a specific exosomal membrane protein [53], suggesting that exosomes derived from cancer cells may play a crucial role in mediating communication between cancer cells and TAMs.

Various interactions have been identified between cancer cells and peritoneal macrophages. For instance, multiple chemokines and growth factors produced by cancer cells enhanced the mobilisation of macrophages into the abdominal cavity [54]. Interestingly, in our study, PCS was first identified as the key transmitter secreted by OC cells to regulate Tim4^+^ TAMs through metabolomic analysis and mass spectrometry, we confirmed that the production of PCS in OC cells was inhibited via the FAK signalling pathway following knock‐down of CD81 expression. Some biological toxicities of PCS have been verified across various cell types. We found that PCS promoted the proliferation of OC cells, leading to exacerbation of OC in mice. Although previous studies showed that PCS had no cytotoxicity toward RAW264.7 cells [55]. PCS exhibited a specific effect on Tim4^+^ macrophages. The mitophagy of Tim4^+^ TAMs was enhanced by PCS, which in turn decreased the proportion of Tim4^+^ TAMs. Hence, after knocking down CD81 expression, the two‐pronged effect of PCS markedly promoted the process of OC, deeming that the level of PCS could be the risk factor in OC for clinical diagnosis. It has been established that PCS combined with Cdh1 to regulate the quantity and mitophagy function of Tim4^+^ TAMs. Cdh1 is an adherent junction protein with a transmembrane region. A few studies suggested that Cdh1 regulated macrophage inflammation; however, the exact molecular mechanisms are yet unknown [56, 57]. We used in vitro experiments to verify the role of Cdh1 in the Bnip3‐dependent mitophagy of macrophages; the downstream signalling pathways need to be explored in the future.

In our study, we observed a decline in the number of Tim4^+^ TAMs coinciding with the remission of OC. Casanova‐Acebes et al. showed that retinoid X receptors deficiency reduced Tim4^+^ TAMs to slow primary ovarian tumour growth [58]. Tim4^+^TAMs impair anti‐tumour CD8^+^ T cells immunity, limiting the efficacy of immunotherapies in these microenvironments [51]. Additionally, analysis of human ovarian cancer ascites revealed that CRIg^high^ macrophages exhibited similarities to murine Tim4^+^ TAMs; notably, ovarian cancer patients with higher CRIg^high^ expression had poorer prognosis [45]. Tim4 protein in peritoneal macrophages can tether apoptotic cells to mediate the efferocytosis of macrophages, hinting that Tim4^+^ TAMs might eliminate apoptotic cancer cells or release immunosuppressive cytokines to regulate immune responses, there by promoting tumour metastasis and growth and even evading treatment‐induced apoptosis with hazardous outcomes [59, 60]. Our study elucidated detailed mechanisms regulating Tim4^+^ TAMs in tumour microenvironment, contributing significantly to our understanding of tumour progression. It is regrettable that the immune function of Tim4^+^ TAMs in OC microenvironment has not been well‐studied in our study, which is worth further study in the future.

Taken together, conjoint analysis of multiomics in our study was applied to discover the vital signalling pathway mediated by CD81 and deepen our understanding of PCS between cancer cells and TAMs. Nevertheless, there are still some improvements in our study in future studies. Although we utilised tumour conditional medium to induce overexpression of Tim4 in macrophages, there may be more ways for in vitro experiments, such as constructing a stable cell line with overexpression of Tim4. As our comprehension of the tumour immune microenvironment deepens alongside technological advancements, clearer directions will emerge for research into the pathogenesis of OC.

## Author Contributions

**Jiali Ni:** data curation (equal), visualization (equal), writing – original draft (equal). **Xiaoying Li:** investigation (equal), methodology (equal). **Yue Wu:** data curation (equal). **Xiaodi Tu:** validation (equal). **Xinxin Zhang:** validation (equal). **Lu Wang:** validation (equal). **Hao Xie:** investigation (equal), methodology (equal). **Yayi Hou:** investigation (equal), supervision (equal). **Huan Dou:** funding acquisition (equal), project administration (equal). **Shuli Zhao:** conceptualization (equal), funding acquisition (equal), project administration (equal), resources (equal).

## Consent

All authors consent to the publication of this study.

## Conflicts of Interest

The authors declare no conflicts of interest.

## Supporting information


**Figure S1.** Validation of transfection efficiency in tumour cells. (A) Relative mRNA expression of CD81 in ID8, A2780 and SKOV3 cells measured by qPCR after transfecting CD81‐siRNA. (B) Relative protein expression of CD81 in ID8, A2780 and SKOV3 cells measured by WB after transfecting CD81‐siRNA. (C) Relative mRNA expression of CD81 in ID8, A2780 and SKOV3 cells measured by QPCR after transfecting pcDNA3.1‐CD81. (D) Relative protein expression of CD81 in ID8, A2780 and SKOV3 cells measured by WB after transfecting pcDNA3.1‐CD81. Data represent the mean scores±SEM. *p < 0.05, **p < 0.01, ***p < 0.001, ****p < 0.0001.
**Figure S2.** CD81 directly modulated proliferation, invasion, and migration of A2780 cells. (A) CCK8 assay showed the effect of silencing CD81 expression on cell growth in A2780 cells compared to the NC group. (B) Representative images of cell colony formation assay showing the effect of silencing CD81 expression on cell growth in A2780 cells compared to the NC group. (C) Annexin V‐PI staining detected by flow cytometry showied the effects of silencing CD81 expression on apoptosis in A2780 cells. (D) PI staining detected by flow cytometry showed the effects of silencing CD81 expression on cell cycle in A2780 cells. (E) Wound healing assay showed the effects of silencing CD81 expression on cell migration in A2780 cells, and representative images were showed. (F) Transwell assay cytometry showed the effects of silencing CD81 expression on cell migration and invasion in A2780 cells, and representative images were showed. (G) CCK8 assay showed the effect of CD81 overexpression on cell growth in A2780 cells compared to the NC group. (H) Cell colony formation assay showed the effect of CD81 overexpression on cell growth in A2780 cells compared to the NC group. (I) Annexin V‐PI staining detected by flow cytometry showed the effects of CD81 overexpression on apoptosis in A2780 cells. (J) PI staining detected by flow cytometry showed the effects of CD81 overexpression on cell cycle in A2780 cells. (K) Wound healing assay showed the effects of CD81 overexpression on cell migration in A2780 cells, and representative images were showed. (L) Transwell assay cytometry showed the effects of CD81 overexpression on cell migration and invasion in A2780 cells, and representative images were showed. Data represent the mean scores±SEM. *p < 0.05, **p < 0.01, ***p < 0.001, ****p < 0.0001.
**Figure S3.** CD81 directly modulated proliferation, invasion, and migration of SKOV3 cells. (A) CCK8 assay showed the effect of silencing CD81 expression on cell growth in SKOV3 cells compared to the NC group. (B) Annexin V‐PI staining detected by flow cytometry showed the effects of silencing CD81 expression on apoptosis in SKOV3 cells. (C) Wound healing assay showing the effects of silencing CD81 expression on cell migration in SKOV3 cells, and representative images were showed. (D) CCK8 assay showed the effect of CD81 overexpression on cell growth in SKOV3 cells compared to the NC group. (E) Annexin V‐PI staining detected by flow cytometry showing the effects of CD81 overexpression on apoptosis in SKOV3 cells. (F) Wound healing assay showed the effects of CD81 overexpression on cell migration in SKOV3 cells, and representative images were showed. Data represent the mean scores±SEM. *p < 0.05, **p < 0.01, ****p < 0.0001.
**Figure S4.** Validation of stably transfection efficiency in ID8 cells. (A) Relative protein expression of CD81 in ID8 cells measured by Western blot after transfecting CD81 shRNA. (B) Median Fluorescence Intensity (MFI) of CD81 detected by flow cytometry after transfecting CD81 shRNA. (C) Relative protein expression of CD81‐associated molecules (p‐PI3K, PI3K, p‐Akt, Akt) in ID8 cells measured by WB after transfecting CD81 shRNA. (D) Immunofluorescence staining of Ki67 and DAPI in the sh‐CD81 cells compared to the NC cells. (E) Transwell assay cytometry showing the effects of stably silencing CD81 expression on cell migration and invasion in ID8 cells, and representative images were showed. Data represent the mean scores±SEM. *p < 0.05, **p < 0.01, ***p < 0.001, ****p < 0.0001.
**Figure S5.** Knock‐down of CD81 expression in OC cells ameliorated metastasis in the murine model of OC. (A) Representative fluorescent images of the organs (liver, spleen, lung, heart, kidney, and tumour) by in vivo imaging system in the peritoneal metastasis murine tumour model of NC and sh‐CD81 groups; and the statistical results of radiance were showed. (B) Representative images of the organs (liver, spleen, lung, heart, kidney, and tumour) in the peritoneal metastasis murine tumour model of NC and sh‐CD81 groups, and the number of tumour nodules was quantified. Data represent the mean scores±SEM. *p < 0.05, **p < 0.01.
**Figure S6.** (A) CCK8 assay showed the effect of PCS with different concentrations on cell growth in ID8, A2780, and SKOV3 cells during 24 h, 48 h, 72 h, and 96 h. (B) Relative protein expression of p‐FAK, FAK in ID8 cells measured by Western blot after transfecting CD81 shRNA. Data represent the mean scores±SEM. *p < 0.05.
**Figure S7.** PCS affected both OC cells and Tim4+ TAMs. (A) Relative protein expression of Tim4 in tumour‐conditioned RAW264.7 cells (ID8‐TCM) compared to the control group measured by Western blot. (B) Relative protein expression of Tim4 in tumour‐conditioned RAW264.7 cells after PCS stimulation measured by Western blot. (C) Purification efficiency of isolating Tim4+ TAMs in ascites detected by flow cytometry. Data represent the mean scores±SEM. *p < 0.05, ***p < 0.001.
**Figure S8.** (A) 62 genes were selected to construct the PPI network in the STRING database. Different coloured lines represented different interactions. (B) Survival curves of other 18 vital genes in ovarian cancer.
**Figure S9.** PCS affected the percentage of immune cells in ovarian cancer. (A) The percentage of Treg cells detected by flow cytometry in the peritoneal metastasis murine tumour model of NC, NC + PCS, sh‐CD81, and sh‐CD81 + PCS groups. (B) The percentage of T cells and PD‐1 expression in T cells detected by flow cytometry in the peritoneal metastasis murine tumour model of NC, NC + PCS, sh‐CD81, and sh‐CD81 + PCS groups. Data represent the mean scores±SEM. *p < 0.05, **p < 0.01.
**Table S1.** Clinicopathological features and demographic information of OC patients.
**Table S2.** Other MS conditions.
**Table S3.** Primer sequences used for the QPCR analysis.

## Data Availability

The datasets used and/or analysed during the current study are available from the corresponding author upon reasonable request.
