# Supplementary material for: CD81 Aggravates Ovarian Cancer Progression via p‐Cresyl Sulfate‐Mediated Mitophagy in Tim4+ Tumour‐Associated Macrophages
Source: J Cell Mol Med. 2025 Jul 2;29(13):e70701. doi: 10.1111/jcmm.70701 (PMC12221806; doi:10.1111/jcmm.70701)
Supplement: Supplementary file 1 — Figure S1. Validation of transfection efficiency in tumour cells. (A) Relative mRNA expression of CD81 in ID8, A2780 and SKOV3 cells measured by qPCR after transfecting CD81‐siRNA. (B) Relative protein expression of CD81 in ID8, A2780 and SKOV3 cells measured by WB after transfecting CD81‐siRNA. (C) Relative mRNA expression of CD81 in ID8, A2780 and SKOV3 cells measured by QPCR after transfecting pcDNA3.1‐CD81. (D) Relative protein expression of CD81 in ID8, A2780 and SKOV3 cells measured by WB after transfecting pcDNA3.1‐CD81. Data represent the mean scores±SEM. *p < 0.05, **p < 0.01, ***p < 0.001, ****p < 0.0001. Figure S2. CD81 directly modulated proliferation, invasion, and migration of A2780 cells. (A) CCK8 assay showed the effect of silencing CD81 expression on cell growth in A2780 cells compared to the NC group. (B) Representative images of cell colony formation assay showing the effect of silencing CD81 expression on cell growth in A2780 cells compared to the NC group. (C) Annexin V‐PI staining detected by flow cytometry showied the effects of silencing CD81 expression on apoptosis in A2780 cells. (D) PI staining detected by flow cytometry showed the effects of silencing CD81 expression on cell cycle in A2780 cells. (E) Wound healing assay showed the effects of silencing CD81 expression on cell migration in A2780 cells, and representative images were showed. (F) Transwell assay cytometry showed the effects of silencing CD81 expression on cell migration and invasion in A2780 cells, and representative images were showed. (G) CCK8 assay showed the effect of CD81 overexpression on cell growth in A2780 cells compared to the NC group. (H) Cell colony formation assay showed the effect of CD81 overexpression on cell growth in A2780 cells compared to the NC group. (I) Annexin V‐PI staining detected by flow cytometry showed the effects of CD81 overexpression on apoptosis in A2780 cells. (J) PI staining detected by flow cytometry showed the effects of CD81 overexpressi [file JCMM-29-e70701-s001.docx]

**Supplementary material**

**Materials and methods**

**UHPLC-OE-MS analysis**

Metabolites extraction

800 μL extract solution (methanol: water=3:1) containing isotopically-labelled internal standard mixture was added after sample was freeze-dried. After 30 s vortex, the samples were freezed and thawed with liquid nitrogen for 3 times. Then the samples were sonicated for 10 min in ice-water bath. Then the samples were incubated at -40 ℃ for 1 h and centrifuged at 12000 rpm (RCF=13800(×g), R= 8.6cm) for 15 min at 4 ℃. The resulting supernatant was transferred to a fresh glass vial for LC/MS analysis. The quality control (QC) sample was prepared by mixing an equal aliquot of the supernatants from all of the samples.

LC-MS/MS analysis

LC-MS/MS analyses were performed using an UHPLC system (Vanquish, Thermo Fisher Scientific) with a UPLC HSS T3 column (2.1 mm × 100 mm, 1.8 μm) coupled to Orbitrap Exploris 120 mass spectrometer (Orbitrap MS, Thermo). The mobile phase consisted of 5 mmol/L ammonium acetate and 5 mmol/L acetic acid in water (A) and acetonitrile (B). The auto-sampler temperature was 4 ℃, and the injection volume was 2 μL. The Orbitrap Exploris 120 mass spectrometer was used for its ability to acquire MS/MS spectra on information-dependent acquisition (IDA) mode in the control of the acquisition software (Xcalibur, Thermo). In this mode, the acquisition software continuously evaluates the full scan MS spectrum. The ESI source conditions were set as following: sheath gas flow rate as 50 Arb, Aux gas flow rate as 15 Arb, capillary temperature 320 ℃, full MS resolution as 60000, MS/MS resolution as 15000 collision energy as 10/30/60 in NCE mode, spray Voltage as 3.8 kV (positive) or -3.4 kV (negative), respectively.

Data analysis

The raw data were converted to the mzXML format using ProteoWizard and processed with an in-house program, which was developed using R and based on XCMS, for peak detection, extraction, alignment, and integration. Then an in-house MS2 database (BiotreeDB) was applied in metabolite annotation. The cutoff for annotation was set at 0.3. OE data with preprocess and normalization was analyzed by PCA and OPLS-DA using SIMCA-P software (version 16.0). Differentially expressed metabolites were selected by univariate analysis (UVA) (P<0.05, VIP>1).

**Detection of PCS through multiple reaction monitoring (MRM) mass spectrometry**

Sample preparation

Methanol, acetonitrile, and water (2:2:1) were added into 200 μL of cell suspension for a total of 1 mL. 50 μL of internal standard was added into the mixture for cell lysis. Cell lysis was performed by ultrasonic shaking at 4 ℃ for a duration of 20 min. Samples were then centrifuged at 16000 g for 10 min, and we collected 800 μL supernatant containing target substance and isotope internal standard (PCS-d7, Cat. NO.: IR-14887, IsoReag, Shanghai, China) and used vacuum drying to concentrate the sample. Finally, the sample was dissolved again using 50 μL mobile phase for mass spectrometry.

Chromatographic conditions

The Waters C18 column (4.6 mm×250 mm, 5 μm) was used, and the protective column was the Waters C18 column (the column temperature was room temperature). The chromatographic conditions included: mobile phase: water (containing 0.1% formic acid): acetonitrile (30:70); flow rate: 0.3 mL/min, 20 minutes per run; sample load: 5 μL; and isocratic elution was taken.

Mass spectrometry (MS) conditions

Ionization mode: electrospray ion source (ESI), negative ion mode; detection method: multi-reaction monitoring (MRM); collision gas (CAD): -2 psi; curtain air (CUR): 30 psi; Gas 1 (GS1): 50 psi; Gas 2 (GS2): 50 psi; Ion spray voltage (IS): -4500 V; Heating temperature (TEM): 500 °C. Other information was listed in the Table. S2.

**Transmission electron microscopy (TEM) analysis**

Tim4^+^ TAMs (2*10^6^/sample) were collected, washed twice with PBS and then fixed with 2.5% glutaraldehyde. Cells were embedded and stained with uranyl acetate/lead citrate. The observation was done using a HT7800/HT7700 electron microscope (Hitachi, Tokyo, Japan).

**In vivo** **bioluminescent imaging**

After mice were weighed and abdomen depilated, they were intraperitoneally injected with d‑luciferin salt solution (150 mg/kg Cat. NO.: D3509, PuSheng, Nanjing, China). When the light signal reaches the strongest stable plateau period after 10-15 min of injection into the body, small animal imaging device (IVIS Lumina XR, PerkinElmer, MA, USA) with isoflurane gas anesthesia were used to bioluminescent imaging for mice, and the whole time was preferably not more than 35 min.

**Data analysis using TNMplot, GTEx, TCGA database and Kaplan–Meier Plotter**

CD81 gene expression was analyzed between normal tissues and ovarian cancer tissues based on TNMplot (<https://tnmplot.com/analysis/>), TCGA, GTEx (<https://gtexportal.org>) databases. The Kaplan Meier plotter (<https://kmplot.com/analysis/>) was used to evaluating the prognostic value of CD81 in ovarian cancer, which embodied in the overall survival (OS) and progression free survival (PFS) of patients with ovarian cancer. The prognostic value of the high and low expression groups was compared according to the hazard ratio (HR), 95% confidence interval (CI), and logrank P value, P<0.05 were considered to have a significant difference.

**Cell transfections**

Mouse CD81 (NC_000073.7) was cloned into the recombinant pcDNA3.1 eukaryotic expression vector (Generay, Shanghai, China), Mouse Cdh1 (NC_000074.7) was cloned into the recombinant pcDNA3.1 eukaryotic expression vector (RiboBio, Guangzhou, China). All plasmids were verified by DNA sequencing. Overexpression of CD81 or Cdh1 plasmid and empty plasmids were transfected into cancer cells with RFect plasmid DNA transfection reagent (BIOG, Changzhou, China) following the manufacturer’s protocols.

Negative control (NC) and CD81-siRNA mimics were procured from RiboBio Co., Ltd. RFect small nucleic acid transfection reagent was used for transfection (mouse CD81-siRNA mimic: 5’-GTACCTCATTGGAATTGCA-3’; human CD81-siRNA mimic: 5’- GGACCAGATCGCCAAGGAT-3’).

**Cell proliferation, cycle, migration, colony formation and apoptosis assay**

Post-seeding the cancer cell suspension were incubated at 100 μL/well for 24 h. The transfection reagent and CD81-siRNA or pcDNA3.1-CD81 were added into the plate, the cells were continued to culture for the corresponding time (24-72h). 10 μL of CCK8 solution (FMS-AP-003, Fcmas, Nanjing, China) were added per well, and cells were incubated for 1-4h. The absorbance values were measured at 450 nm by a microplate reader (Bio Tek, Winooski, VT, USA).

The real-time proliferation of OC cells was detected by the xCELLigence Real-Time Cell Analysis (RTCA) instrument (ACEA Biosciences, Hangzhou, China). Cells were diluted to 1×10^4^ cells/well and were seeded in the E-plate at 100 µL/well. After cell adhesion, different concentrations of metabolite PCS (0-31.25 µg/ml) were added, and the changes of cell index were recorded in real time by RTCA DP analyzer.

For the cell cycle assay, transfected OC cells were harvested, were collected and fixed with 70% ice-cold ethanol for 12 h at 4°C. After washing with PBS, they were incubated with RNase for 1 h on ice and stained with propidium iodide (PI) for 30 min. The cell cycle was measured by flow cytometry immediately.

OC cells were seeded in six well plates at 500 cells/well (ID8 cells), 1000 cells/well (A2780 cells) for incubating overnight. After transfection of CD-siRNA or pcDNA3.1, cells were then washed with PBS and stained with 0.1% crystal violet solution.

OC cells were seeded in twelve well plates at 2×10^5^ cells/well, and were grown to 80% density. Wound lines were scratched vertically to the bottom with a 200 µl pipette tip. After washing with PBS, cells were incubated in fresh medium withour serum, while CD81-siRNA or pcDNA3.1 were transfected into cells. The wound width was recorded by a Nikon Eclipse TS100LED-F microscope (Nikon, Japan).

Cell migration and invasion assay for OC cells were performed by using 24 well transwell plates coated without or with Matrigel. 200 μl of ID8 cells (2×10^4^ cells/well), A2780 cells (5×10^4^ cells/well) were transferred onto the transwell chambers and cultured for 24 h allowing the cells to move through the extracellular matrix to the lower chamber. The cells on the underside of the inserts were fixed with 4 % PFA for 30 min and stained with 0.1 % crystal violet. Five randomly selected fields on the fixed transwell chambers were counted with three repeats and photographed.

Single cell suspension was prepared to cell concentration of 1×10^6^ cells /ml, 100ul cell suspension was taken and Annexin V-FITC was added (Cat. NO.: FMSAV-100, Fcmacs) was incubated without light for 5min and 10ul 7-AAD (Cat. NO.: 00-6993-50, eBioscience, CA, USA) antibody or PI (FMSAV-100, Fcmas) was added and immediately detected by flow cytometry.

**Flow cytometry**

Mouse ascites single-cell suspension was prepared and stained according to different staining schemes. The sample tube was blocked with Fc Blocker (101320, Biolegend, CA, USA) for 10min, then with CD45-Percp CY5.5 (Clone: 30-F11, TONBO Bioscience, CA, USA), CD11b-FITC (Clone: M1/70, BD Pharmingen, CA, USA), F4/80-PE/Dazzle 594 (Clone: BM8, Biolegend)，Tim4-PE/CY7 (Clone: RMT4-54, Biolegend) were stained with Tim4^+^ TAMs. CD45-FITC (Clone: 30-F11, Invitrogen, CA, USA), CD4-APC (Clone: 30-F11, Biolegend), Foxp3-PE (Clone: FJK-16s, Invitrogen) were used to stain Treg cells. CD45-APC (Clone: 30-F11, Biolegend), CD3-AF532 (Clone: 17A2, eBioscience), CD4-PE/Cy5 (Clone: GK1.5, Biolegend), CD8-APC/Cy7 (Clone: 53-6.7, Biolegend), PD-1-FITC (Clone: 29F.1A12, Biolegend) were used to stain T cells. After 30min of dark staining, cells were washed twice with PBS, and detected with flow cytometer (CYTEKTM NL-2000, China).

**HE staining, immunofluorescence and immunohistochemistry**

The omentum tissue in the abdominal cavity of mice was isolated and completely soaked in 4% Paraformaldehyde (PFA) for fixation. Hematoxylin and eosin (HE) staining was used to evaluate the tumor metastasis of the omentum.

Paraffin embedded mouse subcutaneous tumor tissue and stained with specific anti-Ki67 antibody to evaluate the expression level of Ki67 in the tissue. After immunofluorescence staining was completed, the average fluorescence intensity was analyzed by ImageJ.

Sections from ovarian cancer tissues were stained with anti-CD81 antibody (DF2306, Affinity, Changzhou, Jiangsu) to investigate the expression of CD81, respectively. IHC staining of tumor sections was performed according to the manufacturer’s instructions.

**RNA extraction and quantitative real-time PCR (qPCR)**

Total RNA was extracted from tissues and cells using TRIzol Reagent (Invitrogen). We determined relative gene expression values by using the 2^-ΔΔCt^ method, and used glyceraldehyde 3-phosphate dehydrogenase (GAPDH) as an internal control. Primer sequences are presented in Table S3.

**Western blot analysis**

The primary and secondary antibodies were diluted in accordance with the recommended concentration range of the instructions, and incubated overnight in a shaking table at 4℃. The primary antibodies included: anti-CD81 antibody (Cat. NO.:10037S, CST, MD, USA), anti-p-PI3K antibody (Cat. NO.:4228S, CST), anti-PI3K antibody (Cat. NO.:4257T, CST), anti-Akt antibody (Cat. NO.:4691S, CST), anti-Tim4 antibody (Cat. NO.:sc-390805, Santa Cruz, CA, USA), anti-p-FAK antibody (Cat. NO.:381143, ZENBIO, China), anti-FAK antibody (Cat. NO.:r24276, ZENBIO), anti-TOMM20 antibody (Cat. NO.:382451), anti-Cytc antibody (Cat. NO.:250109, ZENBIO), anti-LC3B antibody (Cat. NO.:3868S, CST), anti-Bnip3 antibody (Cat. NO.:R381756, ZENBIO), anti-Cdh1 antibody (Cat. NO.:340341, ZENBIO), anti-β-actin antibody (Cat. No.:BS6007M, Bioworld, MN, USA), anti-β-tubulin antibody (Cat. NO:BS1482M, Bioworld), and the secondary antibodies included: HRP* Goat Anti-Rabbit IgG(H+L) (RS0002, Immunoway, TX, USA), HRP* Goat Anti-Mouse IgG(H+L) (FMS-MS01, Fcmacs). The film was washed with TBST (Tris-buffered saline, 0.1% Tween 20) for 3 times, 10min each time. Finally, minichem^TM^ chemiluminescence imaging system (Sagecreation, Beijing, China) was used to capture images and analyzed gray values by LANE 1D Analysis software.


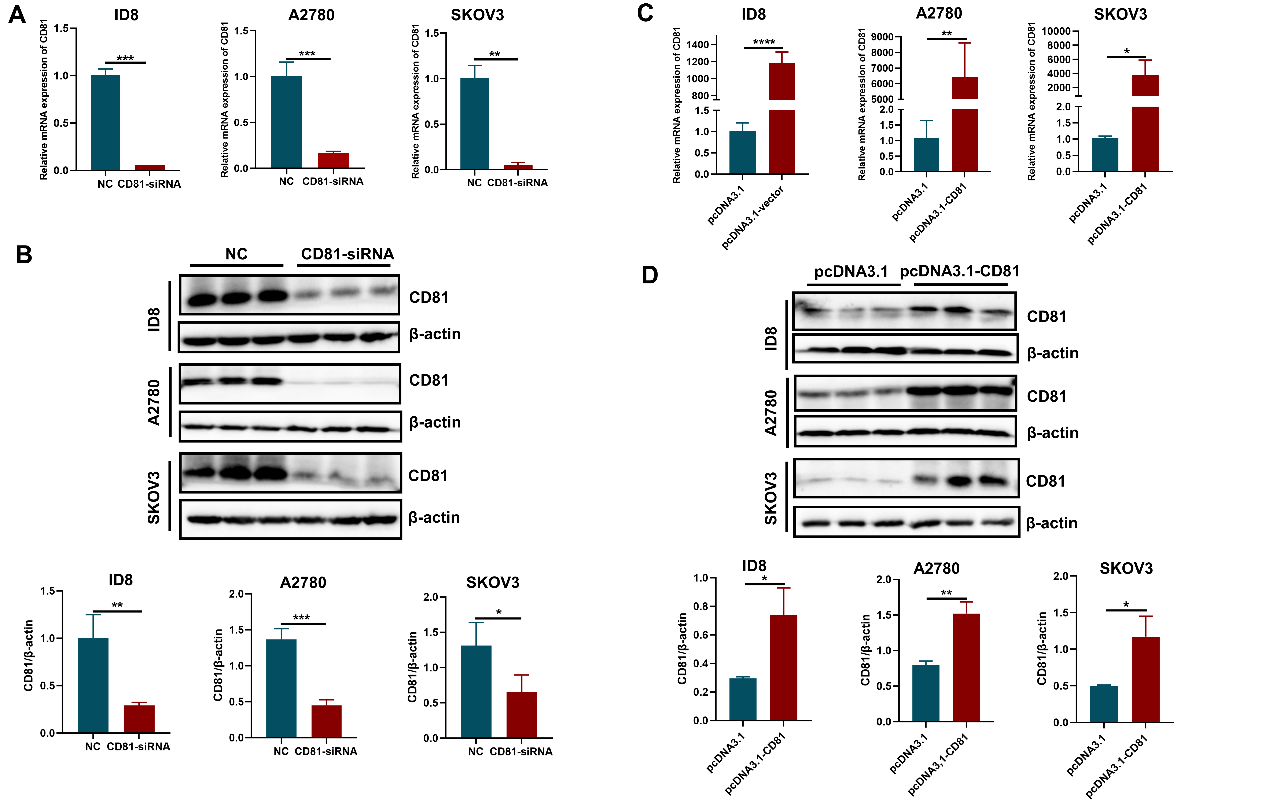


**Supplementary Figure 1.** **Validation of transfection efficiency in tumor cells.** (A) Relative mRNA expression of CD81 in ID8, A2780 and SKOV3 cells measured by qPCR after transfecting CD81-siRNA. (B) Relative protein expression of CD81 in ID8, A2780 and SKOV3 cells measured by WB after transfecting CD81-siRNA. (C) Relative mRNA expression of CD81 in ID8, A2780 and SKOV3 cells measured by QPCR after transfecting pcDNA3.1-CD81. (D) Relative protein expression of CD81 in ID8, A2780 and SKOV3 cells measured by WB after transfecting pcDNA3.1-CD81. Data represent the mean scores±SEM. *P<0.05, **P<0.01, ***P<0.001, ****P<0.0001.


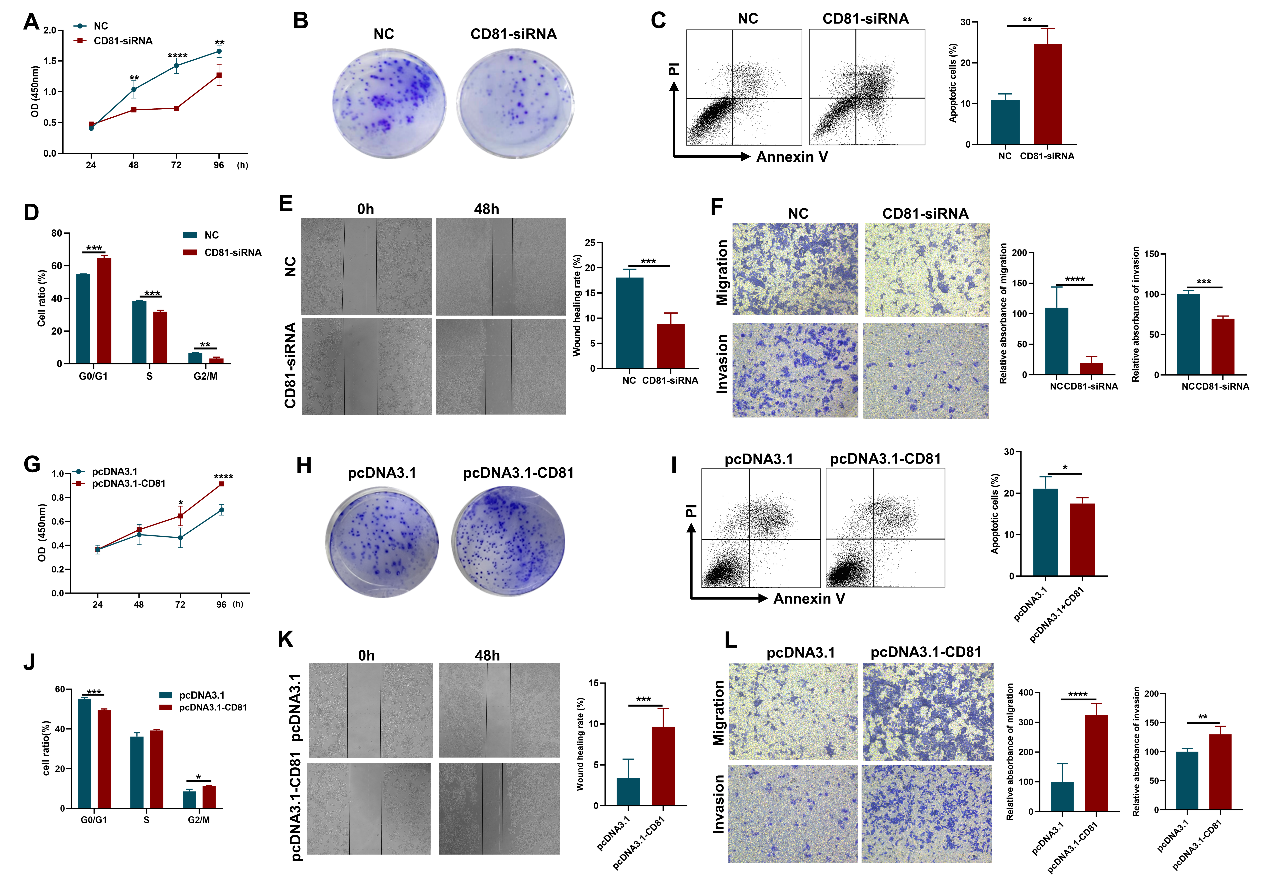


**Supplementary Figure 2. CD81 directly modulated proliferation, invasion, and migration of A2780 cells.** (A) CCK8 assay showed the effect of silencing CD81 expression on cell growth in A2780 cells compared to the NC group. (B) Representative images of cell colony formation assay showing the effect of silencing CD81 expression on cell growth in A2780 cells compared to the NC group. (C) Annexin V-PI staining detected by flow cytometry showied the effects of silencing CD81 expression on apoptosis in A2780 cells. (D) PI staining detected by flow cytometry showed the effects of silencing CD81 expression on cell cycle in A2780 cells. (E) Wound healing assay showed the effects of silencing CD81 expression on cell migration in A2780 cells, and representative images were showed. (F) Transwell assay cytometry showed the effects of silencing CD81 expression on cell migration and invasion in A2780 cells, and representative images were showed. (G) CCK8 assay showed the effect of CD81 overexpression on cell growth in A2780 cells compared to the NC group. (H) Cell colony formation assay showed the effect of CD81 overexpression on cell growth in A2780 cells compared to the NC group. (I) Annexin V-PI staining detected by flow cytometry showed the effects of CD81 overexpression on apoptosis in A2780 cells. (J) PI staining detected by flow cytometry showed the effects of CD81 overexpression on cell cycle in A2780 cells. (K) Wound healing assay showed the effects of CD81 overexpression on cell migration in A2780 cells, and representative images were showed. (L) Transwell assay cytometry showed the effects of CD81 overexpression on cell migration and invasion in A2780 cells, and representative images were showed. Data represent the mean scores±SEM. *P<0.05, **P<0.01, ***P<0.001, ****P<0.0001.


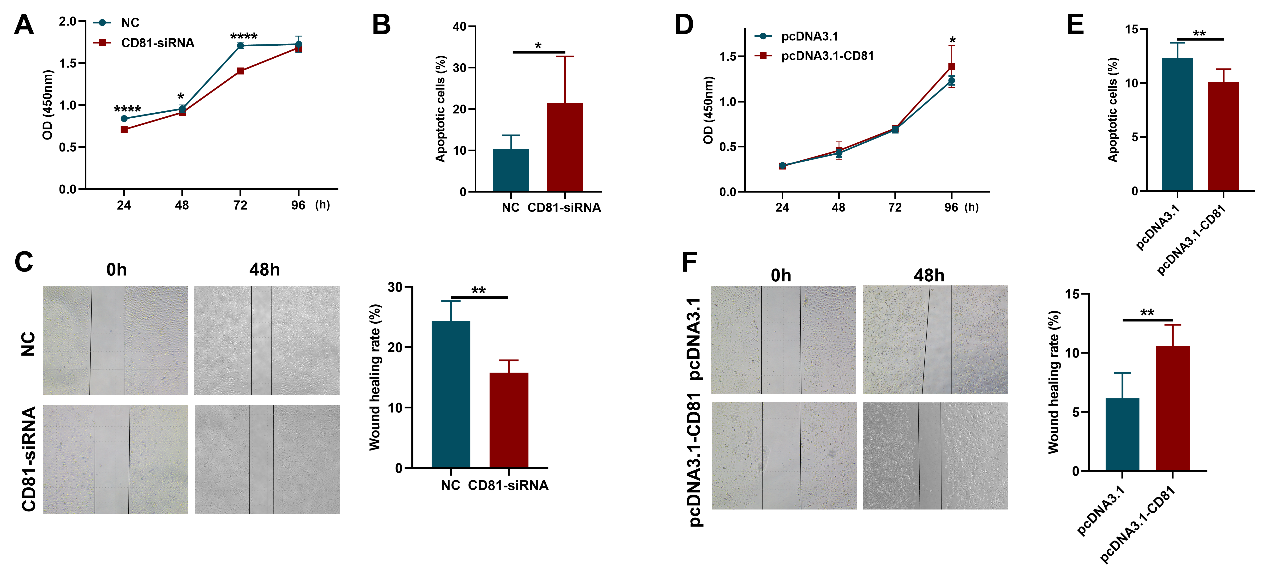


**Supplementary Figure 3. CD81 directly modulated proliferation, invasion, and migration of SKOV3 cells.** (A) CCK8 assay showed the effect of silencing CD81 expression on cell growth in SKOV3 cells compared to the NC group. (B) Annexin V-PI staining detected by flow cytometry showed the effects of silencing CD81 expression on apoptosis in SKOV3 cells. (C) Wound healing assay showing the effects of silencing CD81 expression on cell migration in SKOV3 cells, and representative images were showed. (D) CCK8 assay showed the effect of CD81 overexpression on cell growth in SKOV3 cells compared to the NC group. (E) Annexin V-PI staining detected by flow cytometry showing the effects of CD81 overexpression on apoptosis in SKOV3 cells. (F) Wound healing assay showed the effects of CD81 overexpression on cell migration in SKOV3 cells, and representative images were showed. Data represent the mean scores±SEM. *P<0.05, **P<0.01, ****P<0.0001.


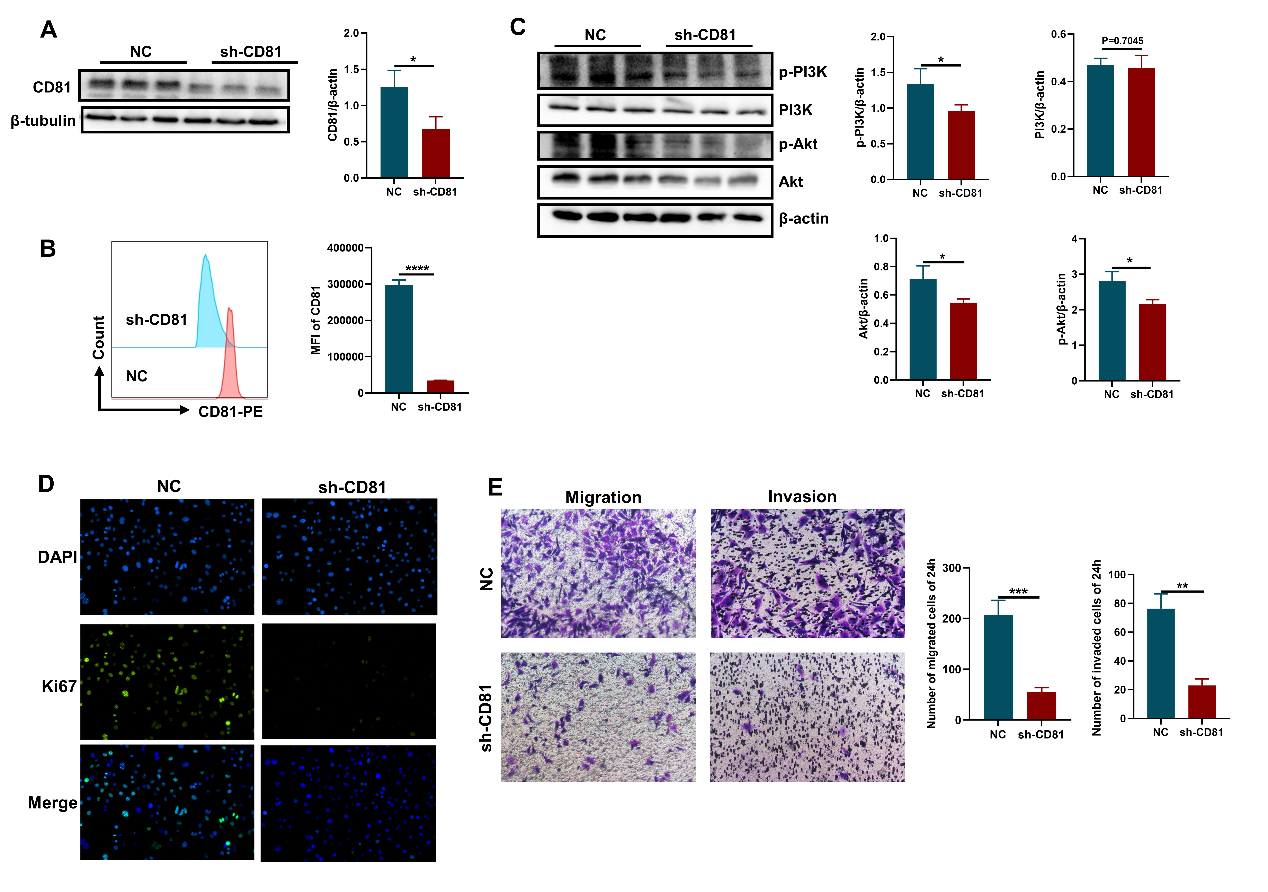


**Supplementary Figure 4. Validation of stably transfection efficiency in ID8 cells.** (A) Relative protein expression of CD81 in ID8 cells measured by Western blot after transfecting CD81 shRNA. (B) Median Fluorescence Intensity (MFI) of CD81 detected by flow cytometry after transfecting CD81 shRNA. (C) Relative protein expression of CD81-associated molecules (p-PI3K, PI3K, p-Akt, Akt) in ID8 cells measured by WB after transfecting CD81 shRNA. (D) Immunofluorescence staining of Ki67 and DAPI in the sh-CD81 cells compared to the NC cells. (E) Transwell assay cytometry showing the effects of stably silencing CD81 expression on cell migration and invasion in ID8 cells, and representative images were showed. Data represent the mean scores±SEM. *P<0.05, **P<0.01, ***P<0.001, ****P<0.0001.


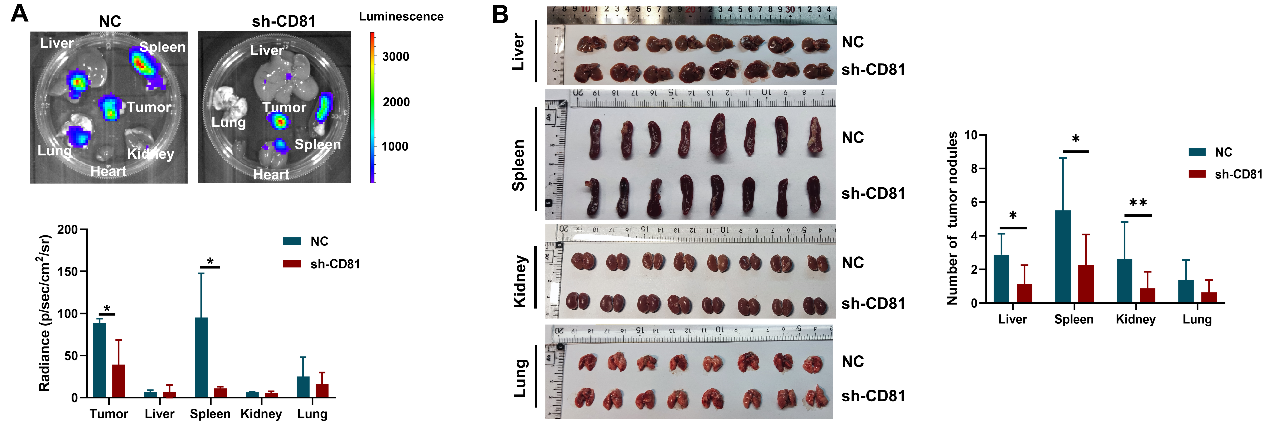


**Supplementary Figure 5. Knock-down of CD81 expression in OC cells ameliorated metastasis in the murine model of OC**. (A) Representative fluorescent images of the organs (liver, spleen, lung, heart, kidney, and tumor) by in vivo imaging system in the peritoneal metastasis murine tumor model of NC and sh-CD81 groups; and the statistical results of radiance were showed. (B) Representative images of the organs (liver, spleen, lung, heart, kidney, and tumor) in the peritoneal metastasis murine tumor model of NC and sh-CD81 groups, and the number of tumor nodules was quantified. Data represent the mean scores±SEM. *P<0.05, **P<0.01.


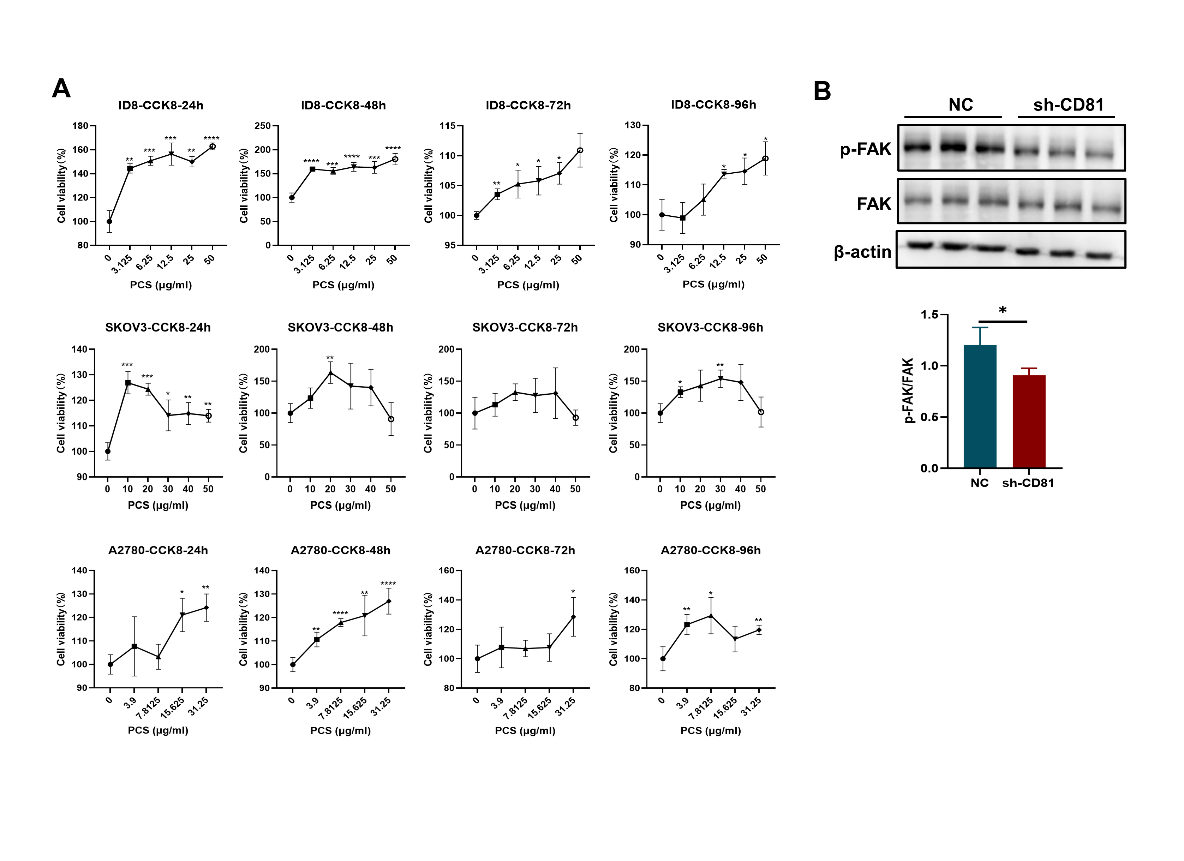


**Supplementary Figure 6.** (A) CCK8 assay showed the effect of PCS with different concentrations on cell growth in ID8, A2780, and SKOV3 cells during 24h, 48h, 72h, and 96h. (B) Relative protein expression of p-FAK, FAK in ID8 cells measured by Western blot after transfecting CD81 shRNA. Data represent the mean scores±SEM. *P<0.05


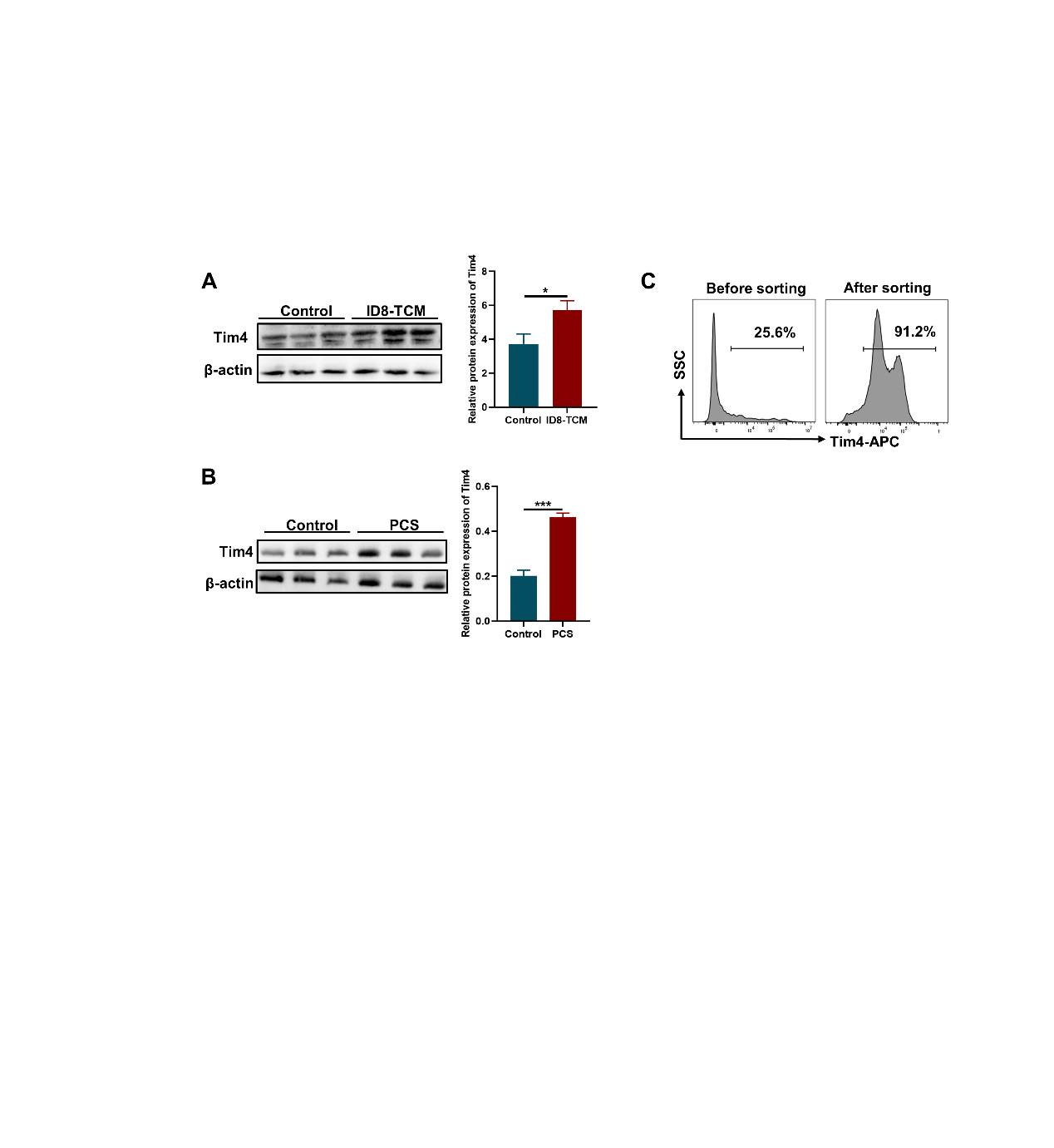


**Supplementary Figure 7.** **PCS affected both OC cells and Tim4^+^ TAMs.** (A) Relative protein expression of Tim4 in tumor-conditioned RAW264.7 cells (ID8-TCM) compared to the control group measured by Western blot. (B) Relative protein expression of Tim4 in tumor-conditioned RAW264.7 cells after PCS stimulation measured by Western blot. (C) Purification efficiency of isolating Tim4^+^ TAMs in ascites detected by flow cytometry. Data represent the mean scores±SEM. *P<0.05, ***P<0.001.


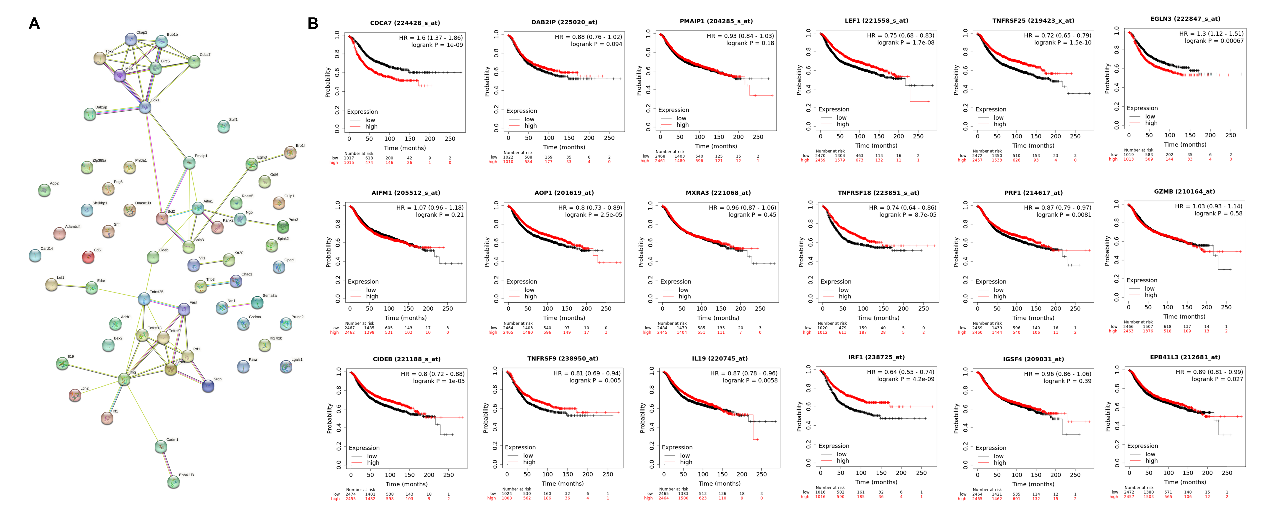


**Supplementary Figure 8.** (A) 62 genes were selected to construct the PPI network in the STRING database. Different colored lines represented different interactions. (B) Survival curves of other 18 vital genes in ovarian cancer.


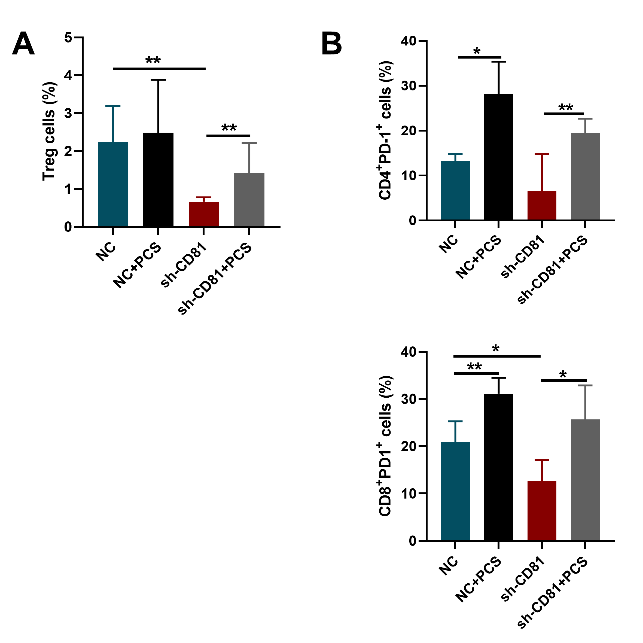


**Supplementary Figure 9.** PCS affected the percentage of immune cells in ovarian cancer. (A) The percentage of Treg cells detected by flow cytometry in the peritoneal metastasis murine tumor model of NC, NC+PCS, sh-CD81, and sh-CD81+PCS groups. (B) The percentage of T cells and PD-1 expression in T cells detected by flow cytometry in the peritoneal metastasis murine tumor model of NC, NC+PCS, sh-CD81, and sh-CD81+PCS groups. Data represent the mean scores±SEM. *P<0.05, **P<0.01.

**Supplementary Table 1. Clinicopathological features and demographic information of OC patients.**

| **Variable** | | **Low CD81 expression (n=17)** | **High CD81 expression (n=17)** | **P value** |
| --- | --- | --- | --- | --- |
| Age | <60 years | 7 | 10 | 0.572 |
|  | ≥60 years | 10 | 7 |  |
| FIGO stage | I - II | 4 | 2 | 0.873 |
|  | III - IV | 13 | 15 |  |

Note：The high and low CD81 expression groups were divided according to the median value of AOD.

**Supplementary Table 2. Other MS conditions.**

| **Target** | **Parameter** | **Condition** |
| --- | --- | --- |
| PCS | Parent ion (m/z) | 186.900 |
|  | Daughter ion (m/z) | 106.900 |
|  | Declustering potential (V) | -130 |
|  | Collision energy (V) | -36 |
|  | Cell exit potential (V) | -12.5 |
| PCS-d7^a^ | Parent ion (m/z) | 193.600 |
|  | Daughter ion (m/z) | 114.600 |
|  | Declustering potential (V) | -130 |
|  | Collision energy (V) | -36 |
|  | Cell exit potential (V) | -12.5 |

a. p-Cresol Sulfate-[D7] Potassium Salt (PCS-d7) is the isotope internal standard to allow absolute quantification.

**Supplementary Table 3. Primer sequences used for the QPCR analysis.**

| **Species** | **Gene** | **Forward primer (5’-3’)** | **Reverse primer (5’-3’)** |
| --- | --- | --- | --- |
| Mouse | GAPDH | AGGTCGGTGTGAACGGATTTG | TGTAGACCATGTAGTTGAGGTCA |
|  | Bnip3 | TCCTGGGTAGAACTGCACTTC | GCTGGGCATCCAACAGTATTT |
|  | Ras | AGTACGTGAGATTCGGCAGC | CACACACTTGCAGCTCATGC |
|  | PI3K | ACACCACGGTTTGGACTATGG | GGCTACAGTAGTGGGCTTGG |
|  | AKT | ATGAACGACGTAGCCATTGTG | TTGTAGCCAATAAAGGTGCCAT |
|  | CDH1 | CAGGTCTCCTCATGGCTTTGC | CTTCCGAAAAGAAGGCTGTCC |
|  | Wnt11 | GCTGGCACTGTCCAAGACTC | CTCCCGTGTACCTCTCTCCA |
|  | Ccn2 | GGGCCTCTTCTGCGATTTC | ATCCAGGCAAGTGCATTGGTA |
|  | Lef1 | TGTTTATCCCATCACGGGTGG | CATGGAAGTGTCGCCTGACAG |
|  | Rassf6 | GACCTTTACCGTATCAGCGAG | GCGTATGGTGTCAGAGTGCT |
|  | Serpine1 | TTCAGCCCTTGCTTGCCTC | TTCAGCCCTTGCTTGCCTC |
|  | Gli2 | CAACGCCTACTCTCCCAGAC | GAGCCTTGATGTACTGTACCAC |
|  | Bmpr1b | CCCTCGGCCCAAGATCCTA | CAACAGGCATTCCAGAGTCATC |
|  | Tim4 | AGAATGTGCGCTTGGAGCTGAG | GGTTGGGAGAACAGATGTGGTC |
|  | Dab2ip | ATCCGAATCAAGGCACGCTAC | CGGTCCACCTCTGACATCAT |
|  | Bcl2 | GTCGCTACCGTCGTGACTTC | CAGACATGCACCTACCCAGC |
|  | Kank2 | CCCACCGTACTCAGTGGAAAC | CTCCAAGGCTCCGTACTGTG |
|  | Egln3 | AGGCAATGGTGGCTTGCTATC | GCGTCCCAATTCTTATTCAGGT |
|  | Prdx3 | GGTTGCTCGTCATGCAAGTG | GGTTGCTCGTCATGCAAGTG |
|  | Tnfrsf25 | CTCACCTTTCTCTTGTGTCCC | CTGGACCCAAAACAGGGGG |
|  | Fasl | TCCGTGAGTTCACCAACCAAA | GGGGGTTCCCTGTTAAATGGG |
|  | Tnfrsf18 | GCCATGCTGTATGGAGTCTCG | CCACTTCCGTTCTGAACCTTG |
|  | Prf1 | AGCACAAGTTCGTGCCAGG | AGCACAAGTTCGTGCCAGG |
|  | Irf1 | ATGCCAATCACTCGAATGCG | TTGTATCGGCCTGTGTGAATG |
|  | Cadm1 | GATCCCCACAGGTGATGGAC | TGATGGTTGCCACTTCTCCTT |
